# Conducting polymer hydrogels for biomedical application: Current status and outstanding challenges

**DOI:** 10.1063/5.0218251

**Published:** 2024-09-24

**Authors:** Matthew S. Horrocks, Kirill E. Zhurenkov, Jenny Malmström

**Affiliations:** 1 Department of Chemical and Materials Engineering, The University of Auckland, 1010 Auckland, New Zealand; 2 MacDiarmid Institute for Advanced Materials and Nanotechnology, 6140 Wellington, New Zealand

## Abstract

Conducting polymer hydrogels (CPHs) are composite polymeric materials with unique properties that combine the electrical capabilities of conducting polymers (CPs) with the excellent mechanical properties and biocompatibility of traditional hydrogels. This review aims to highlight how the unique properties CPHs have from combining their two constituent materials are utilized within the biomedical field. First, the synthesis approaches and applications of non-CPH conductive hydrogels are discussed briefly, contrasting CPH-based systems. The synthesis routes of hydrogels, CPs, and CPHs are then discussed. This review also provides a comprehensive overview of the recent advancements and applications of CPHs in the biomedical field, encompassing their applications as biosensors, drug delivery scaffolds (DDSs), and tissue engineering platforms. Regarding their applications within tissue engineering, a comprehensive discussion of the usage of CPHs for skeletal muscle prosthetics and regeneration, cardiac regeneration, epithelial regeneration and wound healing, bone and cartilage regeneration, and neural prosthetics and regeneration is provided. Finally, critical challenges and future perspectives are also addressed, emphasizing the need for continued research; however, this fascinating class of materials holds promise within the vastly evolving field of biomedicine.

## I. INTRODUCTION

Hydrogels are three-dimensional (3D) networks of hydrophilic polymer chains that can absorb and retain large amounts of water or aqueous solutions within their structure. These versatile materials exhibit a semi-solid consistency, ranging from soft and pliable to more rigid, depending on the specific polymer composition and cross-linking density.^1^ Hydrogels find applications across various fields, including biomedicine,^2^ delivery systems,^3^ tissue engineering,^4^ bioelectronics,^5^ agriculture,^6^ and environmental science.^7,8^ The extracellular matrix (ECM) that surrounds cells in mammalian tissue is itself, by definition, a hydrogel of various proteins and proteoglycans, so it is no wonder that hydrogels are remarkably mechanically similar to the ECM. This makes hydrogels particularly suitable for creating artificial scaffolds to support cell growth and tissue regeneration.^9^ Hydrogels can also be designed to respond to external stimuli, such as temperature, pH,^10^ or light,^11^ allowing for controlled release of encapsulated substances. This responsiveness has led to innovative developments in drug delivery systems, biosensors, and tissue engineering.^12–15^ Additionally, their ability to mimic the mechanical properties of natural tissues has made hydrogels invaluable in creating realistic models of *in vivo* tissues for *in vitro* research and testing of how cells respond to various stimuli.

Conducting polymers (CPs) are polymer materials that possess unique properties typically common in metals, such as conductivity, while still maintaining the beneficial flexible mechanical properties of a polymer.^16^ With their conductivity, facile construction and the flexibility of common polymers, CPs have found use within a vast range of fields.^17–21^ Of relevance for this review are the applications of CPs in the biomedical field, particularly in tissue engineering, biosensing, and drug delivery.^22–24^ CPs are unique in these areas because of their conductive properties in tandem with their flexible behavior and redox properties at mild conditions, allowing for the ability to control various cell behaviors, such as proliferation, differentiation, and adhesion.^25,26^ While CPs excel in their controllability of cells and tissues through electrical stimulation, they are lacking in their biodegradability and mechanical properties, and this has significantly hindered the usage of CPs in practical applications and clinical trials.^27^

Conducting polymer hydrogels (CPHs) were first synthesized in the 1990s by Guiseppi-Elie *et al.*^28^ As the name suggests, they consist of a combination of conducting polymers and hydrogels. Especially within the biomedical field, CPHs boast a variety of advantages over hydrogels or CPs alone by combining the benefits of both constituents. While the conductivity and redox properties of conducting polymers allow them to, for instance, incorporate and release biomolecules for use as drug delivery systems (DDSs) or have sensitive electrical changes for use as biosensors, they suffer in that their mechanical properties are not ideal for cell culture and their poor biodegradability can complicate clinical applications and lead to inflammation.^29,30^ Hydrogels, conversely, are soft and flexible scaffolds utilized in cell culture largely for their excellent mechanical properties. However, although stimuli-responsive hydrogels have been extensively reported,^31,32^ the lack of control and options in doing so limits many potential applications. For instance, as DDSs, achieving controlled and triggered release is complex; as biosensors, the lack of ability for signal transmission is an issue; and for tissue engineering, the lack of conductivity limits many potentially interesting research avenues that take advantage of the known response of cells to electrical fields and the electrical signaling occurring within healthy human tissues.^33,34^ It is clear from the list of advantages of both conducting polymers and hydrogels alone that they would create excellent composite materials that combine these advantages while addressing the deficiencies of each system alone, especially considering that the hydrogel can act as a permeable ion exchange media for conducting polymer actuation. Because of this, recent literature has heavily emphasized utilizing CPH systems for biochemical and biophysical cell studies.

This review summarizes design strategies for fabricating hydrogels and CPs and the design rationale behind combining these materials to create CPHs. This review then explores the primary applications of CPHs within the field of biomedicine: biosensors, drug delivery scaffolds (DDS), and tissue engineering platforms. Other means of developing conducting hydrogels are also discussed in less detail, including carbon nanomaterial-based conductive hydrogels, metal nanomaterial-based conductive hydrogels, and ionic conductive hydrogels.

## II. NON-CPH APPROACHES TO ACHIEVE ELECTRICALLY RESPONSIVE HYDROGELS

**FIG. 1.**
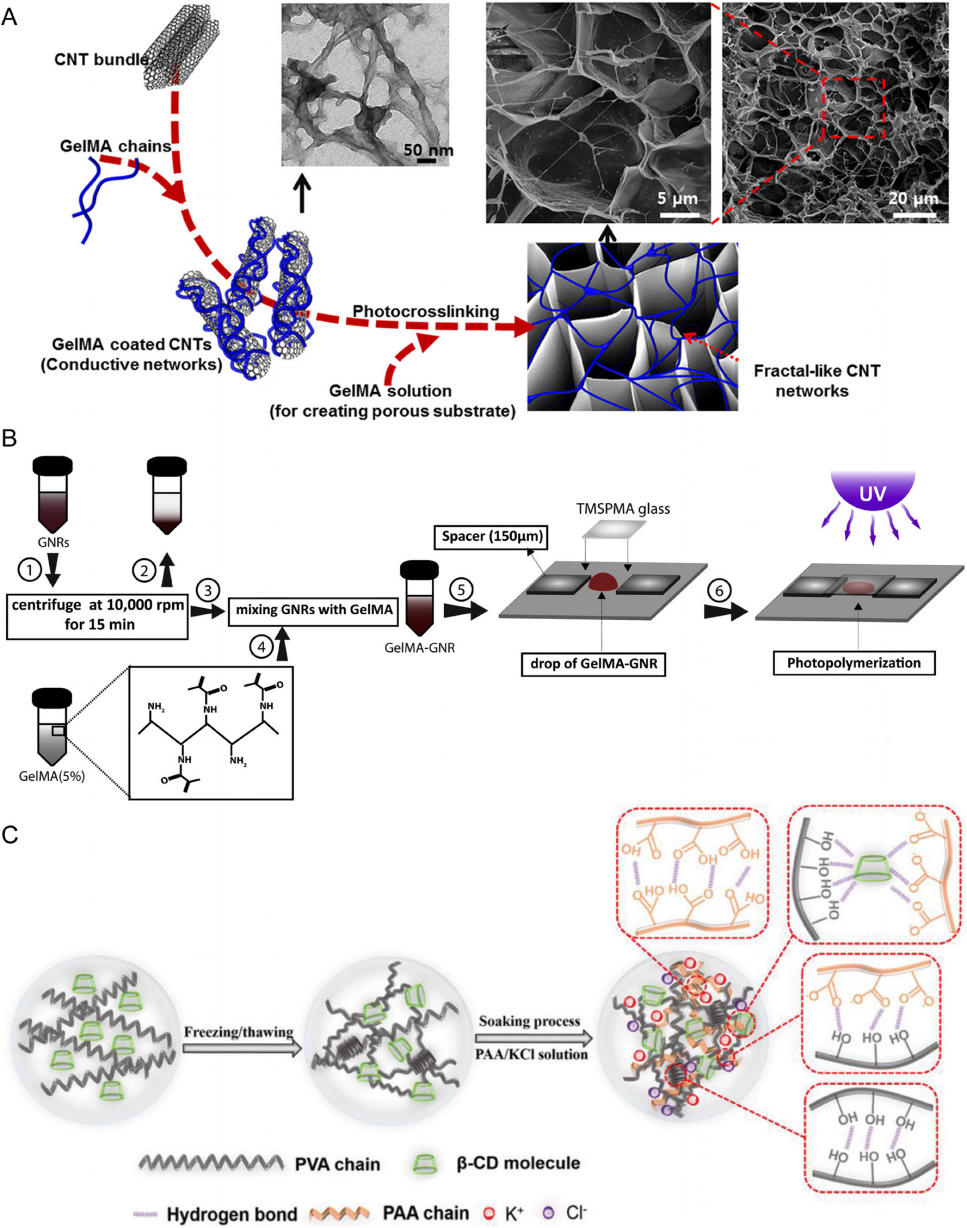
Alternative approaches on fabricating electrically responsive hydrogels. (a) A schematic representation of CNT-incorporated GelMA hydrogel fabrication and its structural features revealed via transmission and scanning electron microscopies. Reprinted/adapted with permission from Shin *et al.*, ACS Nano **7**(3), 2369–2380 (2013). Copyright 2013 American Chemical Society.^41^ (b) Schematics displaying gold nanorod-incorporated GelMA hybrid hydrogel fabrication. Reproduced with permission from Navaei *et al.*, Acta Biomater. **41**, 133–146 (2016). Copyright 2016 Elsevier.^50^ (c) A schematic representation of dual network ionic conductive hydrogel fabrication based on the β-CD/PVA-PAA/KCl system. Reproduced with permission from Yu *et al.*, Macromol. Mater. Eng. **305**(12), 2000475 (2020). Copyright 2020 John Wiley and Sons.^35^

There are many ways conductive hydrogels have been achieved within literature, with each having different properties and applications. Although CPHs and their applications are the primary focus of this review, this section[Sec s3] briefly describes some of the other forms of conductive hydrogels and their applications (Fig. 1). Other reviews exist that provide more detail into these systems.^35–37^

Carbon nanomaterial conductive hydrogels are among these strategies for producing conductive hydrogels. Carbon-based materials have been prominent in electronics-based research due to their high conductivity, stability, and low cost. Carbon nanotubes (CNTs), graphene, and metal carbide/nitride (MXene) are particular types of carbon-based materials that have been explored as composites with hydrogels to produce conductive hydrogels. In the case of CNTs, their conductivity arises from the p electrons of the carbon atoms forming many delocalized π bonds, allowing for the movement of electrons between atoms. These tubular carbon nanoarrays have been dispersed within many hydrogels to offer their conductive properties to the hydrogel system.^38–41^ For instance, Shin *et al.* designed a CNT-incorporated gelatin methacryloyl (GelMA) hydrogel sheet system for cardiac modeling [Fig. 1(a)].^41^ Myocardial tissues cultured on CNT-GelMA showed three times higher spontaneous synchronous beating rates and 85% lower excitation threshold compared to tissues cultured on solely GelMA samples. Improved cell adhesion, organization, and cell–cell coupling were also noted, and the authors attribute this to the electrically conductive and nanofibrous networks formed by CNTs. The system was also demonstrated as a 3D biohybrid actuator, allowing for controllable cyclic contraction/extension, pumping, and swimming actuations. In the case of graphene, its conductivity is owed to the sp^2^ bonding of carbon sheets, which results in free electrons capable of carrying charge. Typically, graphene has poor dispersion within water and, therefore, is unable to disperse uniformly within hydrogels. Graphene is consequently commonly coated with hydrophilic polymers such as polydopamine to increase its dispersion within the hydrogel.^36^ An influential work demonstrating graphene-based conductive hydrogels was conducted by Xu *et al.*, who designed a flexible graphene hydrogel supercapacitor film.^42^ MXenes are a fascinating conductive material that has been widely explored since they were recently initially proposed.^43^ MXenes are 2D nanosheets that display excellent conductivity, hydrophilicity, and tunability, naturally leading to their use for conductive hydrogels.^44–47^ For instance, Liao *et al.* designed a flexible wearable strain sensor involving a polyacrylamide/poly(vinyl alcohol) (PAAm/PVA) hydrogel dispersed with Ti_3_AlC_2_ MXene.^48^ By monitoring resistance changes with very small applied strains, the potential for the system to be utilized as a sensor was confirmed. These results were extended to real-life finger bending and swallowing tests, where clear resistance responses due to the applied strains were noted.

Although not as prominent within literature, metal-based nanomaterials, such as nanowires, nanoparticles, and nanorods, have similarly been dispersed within hydrogels to yield conductive hydrogels. These materials have been utilized extensively, especially for wearable flexible sensors and conductive biomaterial scaffolds, due to their high conductivity, relatively simple fabrication, optical properties, and catalytic properties.^36^ A recent study demonstrated that tungsten or silver microparticles/nanowires can be dispersed within alginate hydrogels for use as conductive biomaterial scaffolds.^49^ The system was shown to have tunable mechanical and electrical properties, and conductivities up to 1000 S m^−1^ were observed using silver nanowires. The low metal content required allowed hydrogel to retain its soft, viscoelastic properties. Another study incorporated gold nanorods into GelMA hydrogels to create more effective engineered cardiac tissue constructs [Fig. 1(b)].^50^ Gold nanorod inclusion was seen to increase the conductivity and stiffness of the hydrogel matrix, as is suitable for cardiac tissue models. Cardiomyocyte studies on the GelMA gold nanorod substrates showed excellent cell retention, viability, metabolic activity, and supported synchronous tissue-level beating. In recent years, liquid metals have also been used as hydrogel fillers to improve conductivity. For instance, Li *et al.* used liquid metal as a filler for a chitosan-based hydrogel.^51^ It was seen that the liquid metal filler toughens the hydrogel, provides excellent antibacterial properties, and provides high conductivity. The hydrogels were also tested as wearable sensors, where human activities could be detected and discerned from subtle motions.

A final prominent way in which conductive hydrogels have been achieved within literature is by using conductive electrolytes rather than conductive compositing additives. These are known as ionic conductive hydrogels.^52^ To achieve this, salts (such as NaCl, KCl, LiCl, CaCl_2_, and FeCl_3_) are included within the electrolyte, and these salts can ionize free metal ions, which can then move freely within the hydrogel with the application of an external voltage.^37^ The movement of ions, and hence the conductivity of the system, can be readily controlled by adjusting the pore size and distribution of the hydrogel.^37^ Furthermore, the composition of the hydrogel can also control the conductivity based on factors such as the electrostatic interaction of the hydrogel matrix and the physical structure of the hydrogel.^53^ Because ionic conductive hydrogels are more similar to ion transport in natural tissue than the electron conductive hydrogels mentioned prior, they have seen particular usage within the fields of wearable sensors and simulated human soft tissue.^44^ Yu *et al.* soaked β-cyclodextrin (β-CD)/PVA hydrogels in poly(acrylic acid) (PAA)/KCl, yielding a dual network ionic conductive hydrogel [Fig. 1(c)].^35^ The conductivity of the system was due to osmotic pressure differences between the PAA/KCl solution and the β-CD/PVA hydrogel, allowing for K^+^ and Cl^−^ diffusion through the hydrogel, and also carboxyl groups present on the PAA phase further contributed to conductivity. The system was reported to maintain the beneficial mechanical properties of the hydrogel, while also demonstrating excellent electrically sensitivity to strain and pressure, making it a promising material for flexible wearable sensors.

## III. DESIGN OF CPHs

### A. Hydrogels

**FIG. 2.**
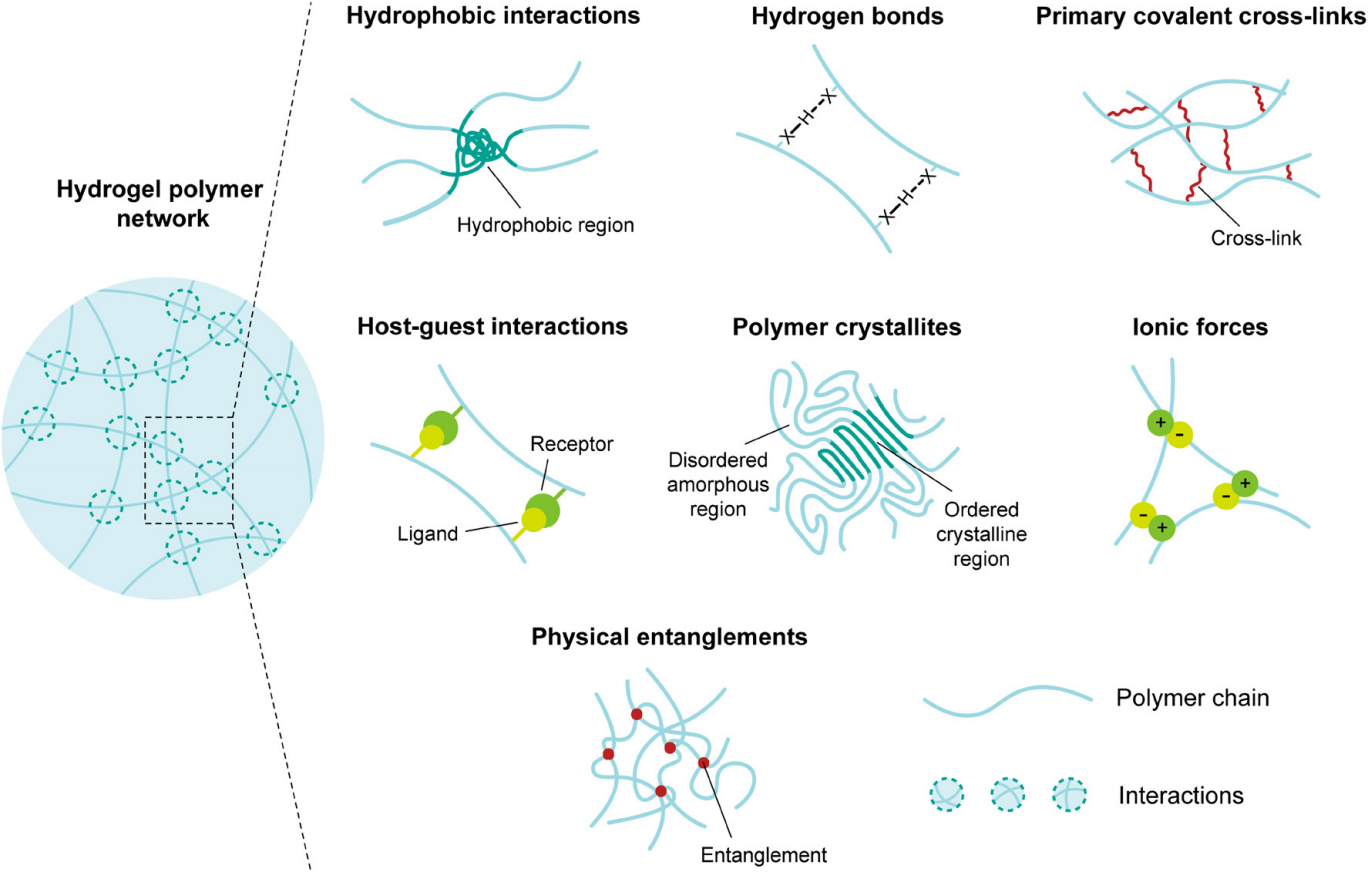
Simplified concepts of hydrogel cross-linking strategies via physical or chemical interactions.

Hydrogels are synthesized through the formation of cross-links between polymer chains that can start from either existing polymers or a monomer solution with an additional polymerizing step. Both covalent and physical means can be employed to achieve this. Physically cross-linked hydrogels rely on non-covalent bonding between polymer chains that are strong enough to prevent hydrogel disassembly in water. Various non-covalent interactions can contribute to this, including polymer chain entanglement, ionic interactions, hydrogen bonds, polymer crystallization, and host–guest interactions.^54^ A summary of the cross-linking strategies discussed here is presented in Fig. 2.

Physical cross-linking by ionic interactions is typically achieved by incorporating ions or ionizable groups into the polymer network. When the polymer comes into contact with an aqueous solution containing ions, these ions interact with the ionizable groups on the polymer chains, forming reversible electrostatic bonds.^55^ These electrostatic interactions create physical cross-links, causing the polymer solution to gel. The strength of the cross-links and the resulting hydrogel properties can be controlled by adjusting factors such as the type and concentration of ions, the pH of the surrounding solution, and the choice of polymer.^1^ Examples of polymers that can cross-link via this method include alginate,^56^ chitosan,^57^ and poly-di(carboxylatophenoxy)phosphazene (PCPP).^58^ Physical cross-linking via hydrogen bonding exploits relatively strong molecular bonds that arise from interactions between a proton and a strongly electronegative atom. When polymers with hydrogen bonding groups, such as hydroxyl or amine groups, are exposed to water, they can participate in hydrogen bonding with similar groups on adjacent polymer chains.^55^ This ultimately forms the structure of the 3D network that can retain water. Hydrogels can also be physically cross-linked through the crystallization of the polymer chains, which requires that they are partially aligned.^59^ Common techniques to achieve this include using freeze–thaw cycles with existing small regions of crystallinity^60^ or by forming stereo-complexes between two enantiomeric polymers.^61,62^ Finally, host–guest interactions are a popular form of physical cross-linking. The “host” is typically a molecule with a large cavity, while the “guest” are molecules with shapes that complement the host.^63^ The geometry of the host and guest molecules is important such that various noncovalent interactions can act to form the cross-links. These interactions include hydrogen bonding, electrostatics, van der Waals forces, and hydrophobic interactions.^64^

Covalent cross-linking, unlike physical cross-linking, is irreversible and involves the induction of covalent cross-links between polymer chains. This process requires cross-linking agents or reactive functional groups within the polymer chains. Covalent cross-linking is widely used in biomaterials, drug delivery, and tissue engineering due to its tunable characteristics and long-lasting stability. Mechanisms involved in achieving the covalent cross-linking of hydrogels include radical polymerization, reaction between functional groups, and cross-linking by high-energy irradiation.

For the radical polymerization formation of hydrogels, radical polymerization occurs on low molecular weight monomers in the presence of cross-linking agents. Radicals are produced by introducing a radical initiator with heat, light, or reductive/oxidative molecules. These free radicals attack unsaturated bonds within the monomers, which in turn propagates polymerization. A cross-linking agent can also be radicalized, which leads to the formation of cross-links between the polymerized chains. This reaction continues to propagate until completion or termination.^65^ Considering the variety of chemical additions, many of which are known to be toxic to cells, it is recommended to wash the resulting hydrogel.^66^ Although free radical polymerization is not controlled, it has been shown to yield a variety of polymer architectures such as chains, grafts, brushes, and star polymers.^67^ Radical polymerization is particularly popular for biomedical applications considering the relatively mild conditions required due to the low amount of initiator species required; however, the rapid and uncontrollable reaction can result in a wide distribution of molecular weights and other inhomogeneities.^65,68^ Chemical cross-linking can also be achieved through reactions between functional groups.^1^ Popular reactions to reach a cross-linked state include click chemistry^69^ and Michael-type addition reactions.^70^ Click chemistry involves the reaction between two groups that readily react, for instance, amine-carboxylic acid, isocyanate-OH, isocyanate-NH, or aldehyde-hydrazide. The cross-linking density of the resultant hydrogel is governed by the quantity of reactive groups in the polymer.^67^ Michael-type addition reactions involve the nucleophilic attack of a nucleophile on a carbon–carbon double bond of an electron-deficient molecule. These reactions form a new carbon–carbon bond, which can be a cross-link between two polymers. The most common Michael-type addition reaction is between thiol and an alkene to form alkyl sulfide.^70^ Although Michael-type reactions provide precisely defined hydrogel networks, they are prone to undesired side reactions and residual unreacted functional groups.^67^ High energy irradiation, such as gamma and electron beams, can also be used for chemical cross-linking of hydrogels.^71^ When this high-energy irradiation interacts with the polymer, free radicals can form via the cleavage of covalent bonds. These free radicals can then react with other polymer chains or themselves, leading to a recombination process forming new covalent bonds. This process can be performed without toxic cross-linking agents. However, residual free radicals are a consideration and can damage biologically active materials that may be present.^1^

### B. Conducting polymers

**FIG. 3.**
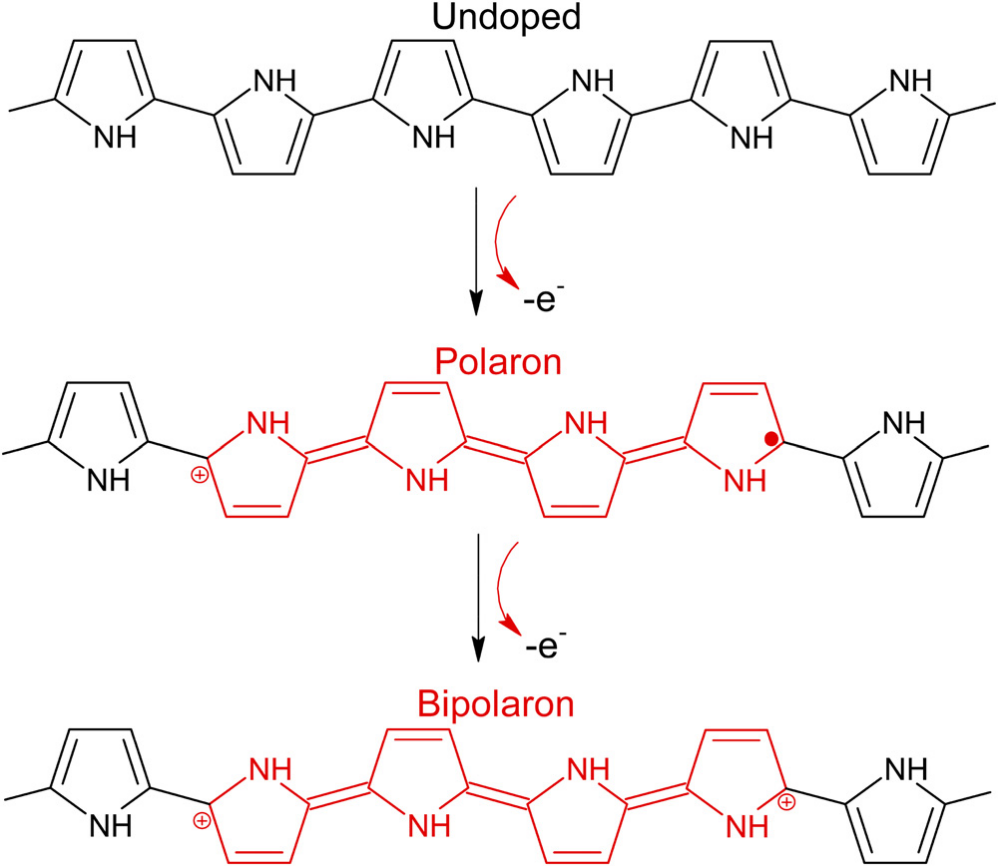
P-type doping process for polypyrrole. Reproduced with permission from Pang *et al.*, Polym. Adv. Technol. **32**(4), 1428–1454 (2021). Copyright 2021 John Wiley and Sons.^76^

CPs were first discovered in 1977 by MacDiarmid *et al.*, who produced polyacetylene doped with iodine to generate a polymer with significant conductivity.^72^ This conductive property arises from the unique structure of CPs, in that they have alternative double- and single-bonded sp^2^ hybridized atoms along the polymer backbone.^18^ Overlapping of p-orbitals gives rise to delocalized π orbitals, which can be either filled (π-bonding orbitals) or empty (π^*^-antibonding orbitals). These different types of π orbitals form the valence and conduction bands within the CP, respectively.^73^ The two energy states of the π orbitals have degenerate energy levels, which ultimately allows charge mobility along the polymer backbone, allowing for the possibility for charge mobility across the CP backbone. Doping is a process, which is also important for enhancing the conductive properties of CPs. In doping, electrons are added (n-type) or removed (p-type) in the polymer backbone. For n-type doping, electron charge carriers are formed when an electron is added to the conduction band, while for p-type doping, hole charge carriers are produced when an electron is removed from the conduction band. The majority of CP systems utilize p-type doping.^74^ When doped, the system's net charge is zero due to the attraction and balancing in charge between the CP backbone and the dopant counterions. This introduces charged polarons into the polymer, and electrons or holes can move from one repeat-unit to nuclei of neighboring units, resulting in charge mobility along the backbone. When this backbone segment is oxidized, the unpaired electron of the polaron is lost, forming a bipolaron, resulting in further increased charge mobility. This process is shown in Fig. 3. The π-bonding orbitals discussed prior enhance the mobility of these charge carriers,^73^ and hence, the overall conductivity of the CP is a function of the potency of the introduction of charge carriers by the dopant and the mobility of the charge carriers.^75^

CP synthesis can be performed via either electrochemical or chemical polymerization. Electropolymerization involves the controlled application of an electric potential between the working electrode and a counter electrode in the presence of a monomer solution. When the voltage is applied, the monomer molecules undergo oxidation or reduction reactions at the electrode surface, leading to the formation of radical species. These radicals then initiate polymerization, where the monomers bond to form a continuous polymer film on the electrode. The electropolymerization process allows for the fabrication of thin, uniform, and adherent polymer coatings with control over the thickness and properties of the resulting polymer film. Many techniques are available to achieve electropolymerization. For instance, potentiostatic polymerization is a particularly popular technique when electropolymerizating CPs^77–79^ and involves applying a fixed potential between the working and reference electrodes while the resulting current is monitored. Potentiostatic polymerization is a versatile method, which allows precise control over the amount of charge passed, and yields well-defined conducting polymer films with relatively smooth surfaces.^80^ Galvanostatic polymerization is a technique where constant current is applied between the working and counter electrodes.^81–83^ This current is typically adjusted to achieve the desired deposition rate and thickness of the CP film.^82^ Galvanostatic polymerization is particularly useful when a specific film thickness or growth rate is desired, as these parameters depend on the current during electropolymerization and the total charge passed, respectively, which are both precisely controlled during galvanostatic polymerization.^80,84^ These films may exhibit variations in thickness and morphology, though, and special care should be taken to avoid overoxidation. Finally, cyclic voltammetry (CV) polymerization involves applying a potential to the working electrode that sweep linearly back and forth within a defined range. The repetitive reduction and oxidation and reduction during the forward sweep and the reverse sweep, respectively, results in CP film growth on the electrode surface.^85^ This technique allows for control over many parameters, such as scan rate, potential range, and number of cycles, and because of this it is particularly effective in controlling film thickness, film morphology, and the properties of the film.^84^

Chemical polymerization can be performed via larger-scale batch production than electrochemical polymerization; however, the reaction occurs over hours rather than minutes.^18^ Electrochemical polymerization can form thin CP films, while chemical polymerization instead typically forms particular suspensions or bulk solids.^18^ The dopant for chemical polymerization is also limited to very small anions, such as chloride and sulfate,^67^ and the conductivity of chemically synthesized CPs is typically lower than that of electrochemically synthesized CPs.^86^

### C. Fabrication of conducting polymer hydrogels

**FIG. 4.**
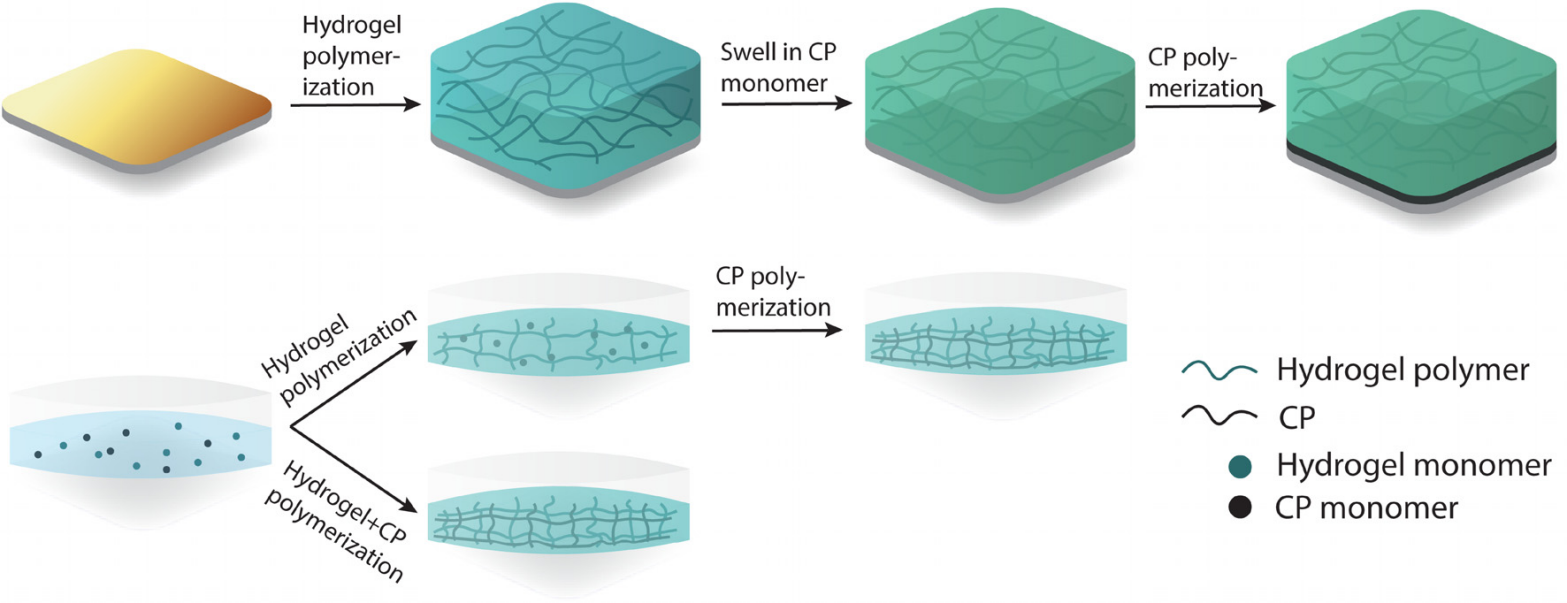
Fabrication pathways for conducting polymer hydrogels.

There are many documented ways of forming CPHs, which can be classified into either polymerization of the CP within a pre-established hydrogel network or direct mixing of the CP and hydrogel monomers with simultaneous polymerization, as shown in Fig. 4. CP polymerization within a pre-established hydrogel network is the most common form of CPH fabrication.^24^ Simply, it involves the typical formation of a hydrogel on a conductive substrate, which is then dried and reswollen in a CP monomer-dopant solution. Polymerization of the CP phase through typically electropolymerization or chemical polymerization results in the final CHP product. When formed via electropolymerization, the electrochemical and mechanical properties are dictated by the type and quantity of the dopant ion and the interaction and dispersion of the CP within the hydrogel matrix.^87^ These parameters can be altered via careful selection of parameters, such as polymerization potential, dopant selection, CP and dopant monomer concentrations, current passed during electropolymerization, and electrolyte pH.^16^ Electrochemical polymerization of CPs within a pre-established hydrogel network has been reported for CPH composite networks, such as GelMA/polypyrrole (PPy),^88^ GelMA/poly(3,4-ethylenedioxythiophene) (PEDOT),^90^ poly(vinyl alcohol)-heparin methacrylate (PVA-Hep-MA)/PEDOT,^90^ alginate/PEDOT,^91^ agarose/PPy,^92^ and poly(dimethylacrylamide-co-4-methacryloyloxy benzophenone-co-4-styrenesulfonate) (PDMAAp)/PEDOT.^93^ The interpenetration of the CP within the hydrogel network is challenging to achieve via electropolymerization due to the propensity of the CP to polymerize near the surfaces of the working electrode surface.^24^ The result is often semi-penetrating CPH networks,^94^ although recent reports have demonstrated complete interpenetration of CP within the hydrogel network. For instance, Bansal *et al.* demonstrated interpenetration of PPy through a GelMA hydrogel via Fourier-transform infrared spectroscopy (FTIR).^88^ Kleber *et al.* also showed a homogeneous distribution of PEDOT through PDMAAp using x-ray photoelectron spectroscopy (XPS).^95^ Chemically polymerized CPHs utilize oxidizing agents such as ferric or persulfate ions on a CP monomer-impregnated hydrogel network.^96,97^ Considering the dispersion of CP monomer during polymerization with this method, an interpenetrating CPH is generated^98^ as there is no driving force toward polymerization at the working electrode; such is the case with electrochemical polymerization. The final form of CPH formation is the polymerization of the CP and hydrogel within the same mixed monomer solution. This is a simple method in which polymerization occurs either simultaneously or in a two-step process chemically or electrochemically.^24^ This methodology has been seen within the literature for GelMA/PEDOT,^99^ PAAm/polyaniline (PANI),^100^ and alginate/PPy systems.^101^ In a similar vein to this methodology, pre-polymerized CP can also been mixed with hydrogel monomers, which are then polymerized.^102,103^

## IV. CONDUCTING POLYMER HYDROGEL APPLICATIONS

### A. Biosensors

Biosensors are analytical devices comprised of a bioreceptor such as enzymes, antibodies, phages, aptamers, or DNA, and a physicochemical transducer. Upon capture of an analyte by the bioreceptor, the transducer converts this recognition event into a measurable signal through electrical, chemical, or optical means.^104^ Since the advent of the first “true” biosensors by Clark in 1962,^105^ they have realized extensive applications within environmental science,^106,107^ biotechnology,^108,109^ medicine,^110^ and food quality control.^111^

While biosensors are widespread throughout not only literature but also commercial application, challenges still remain regarding the design choices to maximize their efficacy for given applications. For instance, the bioreceptors and bio-substrates are non-conductive organic materials. Furthermore, biosensors based on direct electron transfer show poor lifetime and stability.^26^ Finally, it is challenging to find interfacial materials that are biocompatible for immobilization, highly hydrophilic, and will retain the bioactivity of the bioreceptor and analyte.^23^ Both CPs and hydrogels are among the explored interface materials for biosensors, with CPs, in particular, being one of the most popular candidates.^26,112^ In the case of CPs, their use as biosensors has shown improvements in the quantity and flexibility of charge carriers due to the ability to tune both their physical and electrical properties based on their redox or doping/dedoping states.^26^ Furthermore, the conducting mechanism of CPs can involve both electron and ion charge carriers.^23^ CPs generally also maintain a favorable surface for enzyme binding, as well as acting as an effective connection with typical metallic or carbon biosensor electrode materials.^113^ Hydrogel-based biosensors have been explored far less due to their obvious limitation of being generally non-conductive.^114^ They do, however, boast desirable properties for biosensor applications. For instance, they are excellent for immobilizing biological recognition species and are superbly biocompatible due to their ECM-mimetic nature.^115–117^ In pioneering work exploring CPHs as biosensors, which combine the benefits of CPs and hydrogels, it was noted that CPHs make excellent biosensor electrodes because of the electron transfer capabilities of the CP phase, the nanoporous 3D hydrogel structure offering large surface area with low diffusion distances, and excellent biocompatibility.^118,119^ This biocompatibility provided by the hydrogel phase is particularly relevant for implantable sensors, although autonomous CPH sensor devices have also been explored within the literature.

Biosensors can be categorized based on their transduction method into electrochemical, optical, piezoelectric, and thermometric biosensors.^120^ CP- and CPH-based biosensors fall under electrochemical biosensors. In electrochemical biosensors, reactions between the immobilized biomolecule and the analyte produce or consume ions or electrons.^121^ The result of this is a measurable change in the electrical properties, which could be a change in current (amperometric), a change in potential (potentiometric), or a change in the conductive properties (conductometric).^122^ For the case of CP biosensors, this signal transduction can look one of many ways. For instance, with simple small molecules, diffusion of the molecule into the CP can cause reactions altering its doped state.^123,124^ For larger molecules, biomolecule binding to the CP interface yields large signals, and this can be due to many reasons. First, binding can change the potential distribution surrounding the interface, both at the fringe of the solution and the CP, which causes a change in impedance.^125^ Alternatively, ion exchange can occur across the interface, leading to localized dopant concentration changes within the CP near the interface.^126^ Surface charges of adsorbed biomolecules can also increase the local electric field across the interface, which creates areas within the CP where charge carriers are trapped. Finally, additional ions may adsorb into or absorb onto the CP as a result of biomolecule binding, and these ions interact with charge carriers within the CP.^126^

**FIG. 5.**
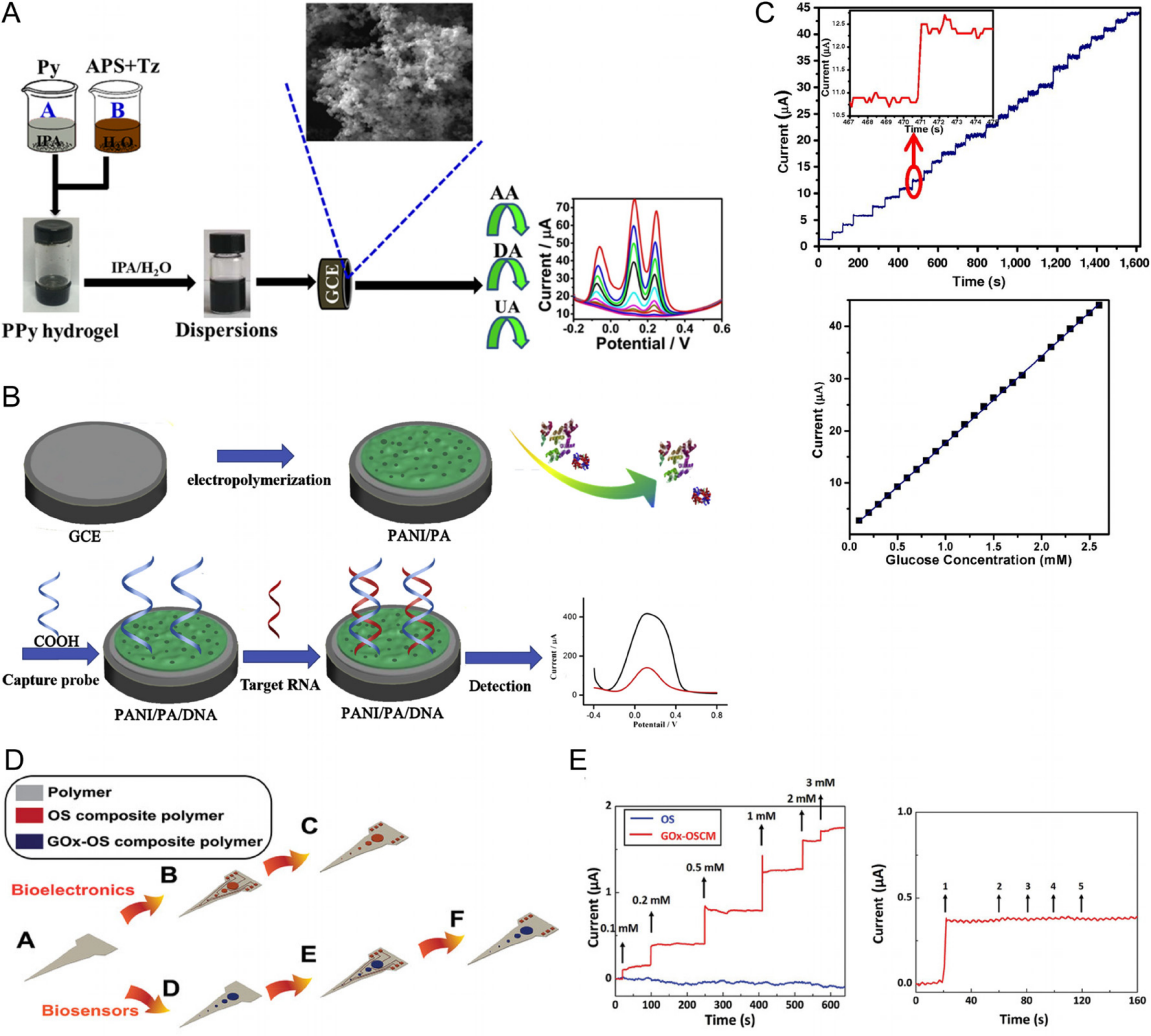
Conducting polymer hydrogel approaches as biosensors. (a) PPy hydrogel preparation and usage as an ascorbic acid, dopamine, and uric acid biosensor. Reproduced with permission from Wang *et al.*, J. Electroanal. Chem. **832**, 174–181 (2019). Copyright 2019 Elsevier.^127^ (b) Fabrication and response of a PANI/PA RNA targeting biosensor. Reproduced with permission from Yang *et al.*, Anal. Chim. Acta **1124**, 104–112 (2020). Copyright 2020 Elsevier.^131^ (c) Amperometric response for a GO_x_-PANI enzymatic biosensor with stepwise 0.1 mM addition of glucose in PBS, and the corresponding calibration plot. Reproduced with permission from Pan *et al.*, Proc. Natl. Acad. Sci. U. S. A. **109**(24), 9287–9292 (2012). Copyright 2012 National Academy of Sciences.^132^ (d) A schematic representation of hybrid Michigan-style microelectrode fabrication using MPL-based technology. (e) Left: Amperometric response of organic semiconductor (blue) and glucose-oxidase-encapsulated OSCMs (red) to successive glucose addition. Right: Amperometric response of glucose-oxidase-encapsulated OSCMs to the addition of 1—glucose, 2—acetaminophen, 3—ibuprofen, 4—ascorbic acid, and 5—urea. Reproduced with permission from Dadras-Toussi *et al.*, Adv. Mater. **34**(30), 2200512 (2022). Copyright 2022 John Wiley and Sons.^102^

Conducting polymer hydrogels have been utilized as sensors for a variety of different analytes within the literature (Fig. 5). For instance, Wang *et al.* developed a PPy hydrogel by utilizing the azo dye tartrazine as a cross-linker and the dopant for PPy for sensing of ascorbic acid, dopamine, and uric acid [Fig. 5(a)].^127^ This is a unique and atypical form of CPH that is often seen when CPHs are used as biosensors, wherein the CP also acts as the hydrogel through cross-links formed between CP chains by a cross-linker, which is, in this case, tartrazine. This PPy hydrogel was dispersed onto a glassy carbon electrode. Using CV and square wave voltammetry (SWV), the unique oxidation peaks of ascorbic acid, dopamine, and uric acid were able to be detected simultaneously, yielding lower detection limits of 1.283, 0.044, and 0.046 *μ*M L^−1^. These analytes were also detected in a real sample analysis of urine. Other CPH systems that detect dopamine have also been developed, such as aptamer-coupled PPy/agarose CPHs^128^ or PEDOT:poly(styrenesulfonate) (PSS) CPHs.^129^ In a recent study, Yang *et al.* extended the capabilities of CPH systems to sense dopamine and showed *in situ* electrochemical cell sensing by monitoring the dopamine released by PC12 cells.^130^ In this study, a PEDOT:PSS CPH was designed via novel preparation methods that bypass the typical toxicity concerns of PEDOT:PSS, which involved cross-linking PEDOT:PSS with different, positively charged CPs (such as pyrrole, aniline, and indole) as cross-linkers. The PEDOT:PSS was prepared on an ITO glass electrode, and gold nanoparticles were electrodeposited within. PC12 cells were then incubated atop the biosensor and stimulated with K^+^ to stimulate dopamine release. An amperometric response was then able to be detected from the biosensor, which increased with increasing K^+^ concentration. This system demonstrates how CPH biosensors can be utilized for sensing 3D cell culture models. Aside from dopamine, other molecules have been sensed using CPH biosensors. For instance, Yang *et al.* assembled a PANI/phytic acid (PA) CPH capable of mRNA detection with an impressive lower detection limit of 0.34 fM [Fig. 5(b)].^131^ For this, PANI/PA was electrochemically deposited onto a glassy carbon electrode and coated with a DNA probe, where PA acted as a cross-linker between PANI chains. Redox currents were utilized as sensing signals to sense DNA/RNA hybridization reactions. The system also demonstrated excellent electrochemical properties and antifouling characteristics. Glucose biosensors have also been developed, for instance, Bao *et al.* constructed a PANI CPH again using PA as the gelator and dopant [Fig. 5(c)].^132^ The biosensor works by immobilizing glucose oxidase (GO_x_) on the CPH and monitoring the enzymatic reaction between GOx and glucose, wherein the detected current increases with increasing glucose concentration. The system noted fast glucose detection times (∼0.3 s) and high sensitivity (∼16.7 *μ*A mM^−1^). Another system developed by Dadras-Toussi *et al.* utilized multiphoton lithography (MPL) to 3D print a resin composed of photopolymer poly(ethylene glycol) diacrylate (PEGA) and PEDOT:PSS, referred to as an organic semiconductor microelectronic device (OSCM) [Fig. 5(d)].^102^ Glucose-oxidase-encapsulated OSCMs demonstrated highly sensitive glucose sensing capabilities, with high sensitivity (232.9 ± 22.5 *μ*A × 10^−3^ M^−1 ^cm^−2^), good specificity, and high reproducibility [Fig. 5(e)].

Another category of biosensors that CPHs have seen wide use in is wearable sensors. Wearable sensors typically serve the purpose of monitoring disease-related signals, which offers huge convenience for patients attending regular and expensive diagnostic hospital visits.^133^ CPHs have garnered traction in this field due to their favorable mechanical properties, biocompatibility, and conductivity. Pressure or strain on a CPH can densify the network, which changes the resistance or conductivity, and because of this, the majority of research on CPHs for wearable sensors has focused on utilizing them as pressure and strain sensors.^134^ Other recent reviews extensively cover these CPH wearable sensors.^135–137^ In short, the physiological signals that CPH wearable sensors have been employed to monitor within literature include mechanical human movements (fingers, wrist, knees, etc.),^138–142^ pulse signals,^143^ and breathing.^143^

### B. Drug delivery

**FIG. 6.**
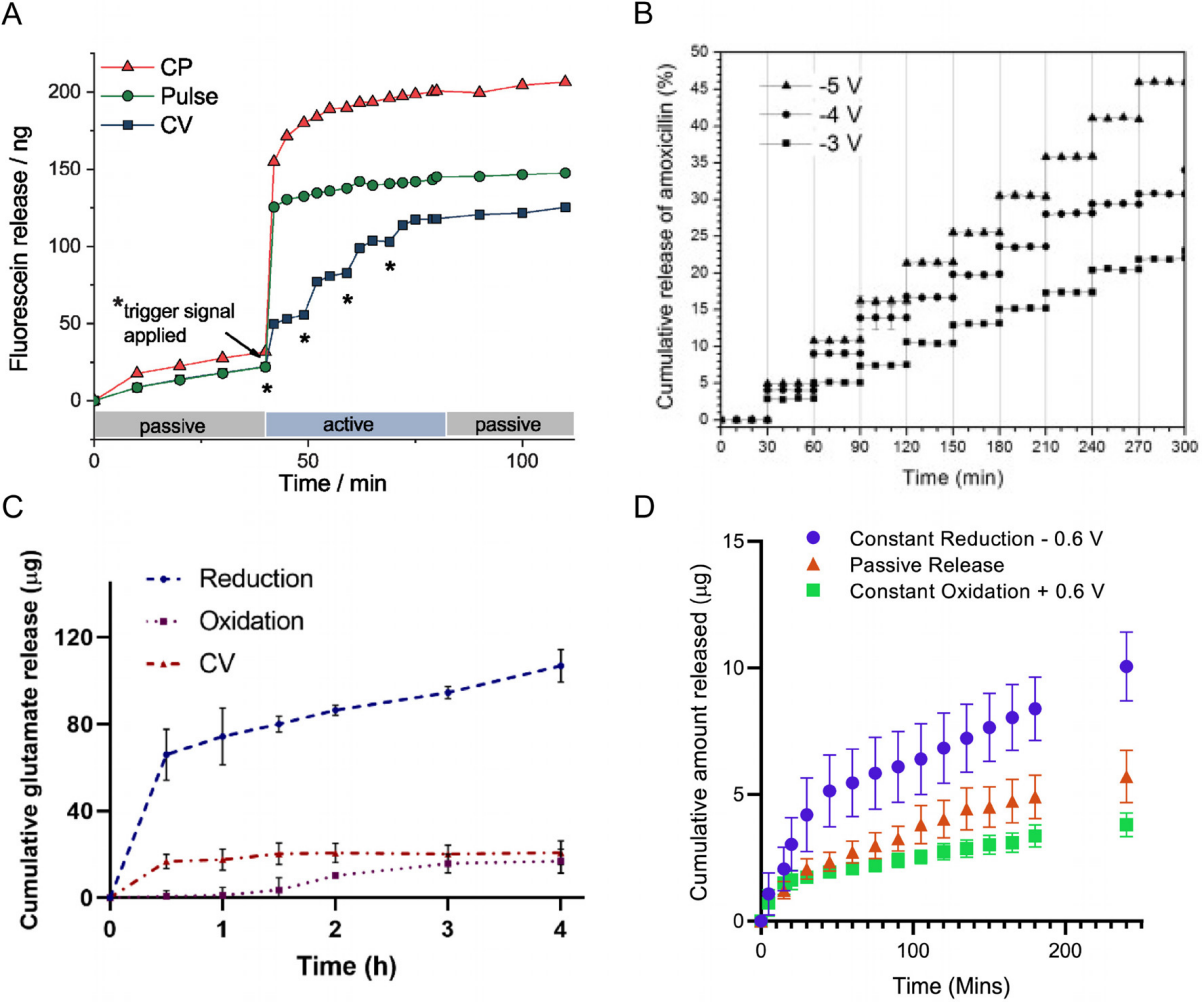
Electrically controlled release of drugs from different CPH composites. (a) Fluorescein release from PDMAAp/PEDOT CPHs for continuous potential (red, −0.5 V), a 60 s pulse (green, −0.5 V), and CV [blue, three cycles of ±0.5 V applied at trigger point (*)]. Reproduced with permission from Kleber *et al.*, Adv. Healthcare Mater. **8**(10), 1801488 (2019). Copyright 2019 John Wiley and Sons.^93^ (b) Amoxicillin release from PAAm/PANI CPHs under −3, −4, and −5 V constant potential stimulation for 1 min, applied every 30 min. Reproduced with permission from Pérez-Martínez *et al.*, React. Funct. Polym. **100**, 12–17 (2016). Copyright 2016 Elsevier.^100^ (c) Glutamate release from GelMA/PPy CPHs under −0.6 V constant potential, compared to when oxidized under +0.6 V constant potential and with CV sweeps (±0.6 V at 100 mV/s). Reproduced with permission from Bansal *et al.*, Acta Biomater. **137**, 124–135 (2022). Copyright 2022 Elsevier.^88^ (d) FITC-BSA release from GelMA/PEDOT CPHs at −0.6 V constant potential compared to passive release with no applied potential, and oxidation at +0.6 V constant potential. Reproduced with permission from Cheah *et al.*, Acta Biomater. **158**, 87–100 (2023). Copyright 2023 Elsevier.^89^

CPs have seen wide use within literature as DDSs by exploiting the reversible redox properties of CPs, allowing for the electrically actuated intake and release of charged molecules. Controlled release DDS systems offer advantages over conventional therapies in that they can provide a maintained concentration of drugs within the effective dosage levels over prolonged periods.^144^ The primary issue with CPs as DDSs is their mechanical mismatch with natural tissues, leading to complications with both *in vitro* research and *in vivo* research and application.^29^ This is especially relevant due to the localized release these systems aim to achieve, which means typically these DDSs will be interfacing directly with cell cultures or tissues. The use of CPHs can logically address this shortcoming by preserving the drug loading and release capabilities of the CP, while introducing the favorable mechanical environment of hydrogels. Inclusion of the hydrogel phase is that CP DDSs is still a relatively unexplored area but has been utilized for the delivery of antibiotic drugs,^100^ anti-inflammatory drugs,^93^ proteins,^89^ glycosaminoglycans,^145^ and neurotransmitters.^88^ PPy, PANI, and PEDOT are the popular CP choices for delivery applications. A comprehensive list of systems that have employed CPHs as drug delivery devices is presented in Table I, and examples of drug release from CPHs are presented in Fig. 6.

**TABLE I.** Summary of electrically controlled CPH drug release systems.

CP	Hydrogel	Dopant/oxidant	Drug	Loading	Release	Ref.
PANI	PAAM	HCl	Safranin	CPH was soaked in safranin solution for one day before passive loading.	Constant potential of −0.1 V, then +0.4 V, or constant potential of −0.2 V, then +0.6 V.	94
PANI	PAAM	APS	Amoxicillin	A PANI suspension was mixed with amoxicillin for loading into the CP, then this was incorporated into the PAAM precursor solution.	Constant potentials from −3 to −5 V for 1 min every 30 min.	100
PANI	PAAM	HCl	Tetracycline	CPH was soaked in tetracycline solution for one day for passive loading.	Constant potential of −0.2 V, then +0.6 V.	146
PANI	PVA	⋯	Indomethacin	Indomethacin included with hydrogel precursors during polymerization, and PANI was added to the hydrogel.	0.3–5 V constant potential for 1 min 4 times.	147
PANI	Collagen	APS	Hydrocortisone	CPH was soaked in a hydrocortisone PBS solution for one day for passive loading.	Passive release measured with no applied potential. Active release measured with constant potential of 3 and 1.5 V.	148
PEDOT	GelMA	PSS	5-Fluoruoracil	CPH was soaked in 5-fluoruoracil for three days for passive loading.	Constant potential of +1.5 V.	99
PEDOT	PDMAA	PSS	Dexamethasone	CPH was soaked in Dex for 12 h for passive loading. CPH was subjected to +0.6 V within a Dex solution for active loading into the CP phase.	Constant potential of −0.5 V for 60 s, or 5 CV cycles from 0.5 to +0.8 V at a 100 mV/s scan rate.	93
PEDOT	Pectin	APA	Ibuprofen	Ibuprofen was included in the polymer precursor solution for loading during hydrogel polymerization. PEDOT was polymerized within the hydrogel.	Constant potential ranging from 0 to +5 V.	149
PEDOT	GelMA	*p*TS	FITC-BSA	The CP was electropolymerized on a substrate carrying a preformed hydrogel from a FITC-BSA, *p*TS, EDOT solution.	Constant potential of −0.6 V and a biphasic pulse of 0.1 or 0.01 Hz.	89
PEDOT	Poly(γ-glutamic acid)	DBS/APS	Curcumin	PEDOT/curcumin was made via emulsion polymerization and loaded into the hydrogel phase.	−0.5 V every 15 min for 24 h.	150
PPy	PEGDA/PAAM	Dexamethasone	Dexamethasone	PEGDA/PAAM was polymerized with Dex present, and PPy was chemically polymerized within this hydrogel.	CV from −0.1 to +0.5 V at a scan rate of 100 mV/s.	151
PPy	GelMA	Glutamate	Glutamate	PPy with glutamate as a dopant was electropolymerized on a substrate with pre-existing hydrogel.	Constant potential of −0.6 V, or CV cycles between −0.6 and +0.6 V for 4 h.	88
PPy	PAAM/Chitosan	FeCl_3_	Dexamethasone	Dex was included in the polymer precursor solution for loading during hydrogel polymerization. Py was polymerized within the hydrogel in the following step.	Constant potential of −1 and −3 V.	152
Poly(phenylene vinylene)	PAAM	Salicylic acid	Salicylic acid	PPV CP particles were made by oxidizing PV with H_2_O_2_ with salicylic acid as a dopant and vacuum drying. These were then mixed with the hydrogel precursors to form the CHP.	Constant potentials of 0, +0.01, +0.03, +0.05, +0.07, +0.09, and +0.1 V.	153

Small molecule drugs are the most common substance released using CPH systems due to physical barriers when attempting to incorporate larger molecules within the relatively dense CP phase.^30^ Kleber *et al.*^93^ used a PDMAAp/PEDOT CPH system for a comprehensive investigation of the electrically triggered release of fluorescein as an anionic model drug and translated this into demonstrating dexamethasone release, which is an anionic anti-inflammatory drug. The passive release of fluorescein from the PDMAAp hydrogel component (1.2 *μ*m thick, dry) was first analyzed, which showed 1.6 ± 0.4 *μ*g cm^−2^ of fluorescein released over 28 days. Totally, 89% of this release was noted over the first 140 min, indicating burst diffusive release. Active release of fluorescein was explored by applying a physiologically safe −0.5 V potential, which is a feat some CPH systems cannot achieve. By holding this reducing potential for 60 s, fluorescein release was nearly tenfold greater for PDMAAp/PEDOT samples over 140 min compared to PDMAAp/PEDOT when stored in PBS for 28 days with no applied potential. Release of fluorescein via CV between −0.5 to +0.5 V was also noted, yielding a staircase release profile. The study further characterized the release capabilities of the system by reloading the CP with fluorescein multiple times via re-oxidation, and release was observed over four release/reload cycles. When replacing fluorescein with dexamethasone, 169.6 ± 7.2 *μ*g of dexamethasone was released after 10 min of electrical stimulation. Future studies of this system would utilize the proven ability to release a pharmaceutical drug in cell studies, which is where the importance of the hydrogel phase would become apparent. Pérez-Martínez *et al.* demonstrated the release of an antibiotic drug, amoxicillin, from a PAAm/PANI CPH.^100^ PANI nanofibers were synthesized via chemical polymerization of aniline, and amoxicillin was loaded onto PANI through a mixing process. These amoxicillin-loaded PANI nanofibers were then incorporated into a polyacrylamide hydrogel for electrically controlled drug release experiments. Release of amoxicillin was electrically stimulated via constant potentials of −3, −4, and −5 V. The release of amoxicillin in this instance is a unique case where the drug carries net zero charge at neutral pH, so the release mechanism in this instance was instead the contraction of the CP due to mass transport from PANI to the electrolyte.

While less common, biomolecules other than pharmaceutical drugs have been released from CPH systems within literature. For instance, a recent study by Cheah *et al.* demonstrated the release of fluorescently labeled bovine serum albumin (BSA) protein from a GelMA/PEDOT CPH.^89^ BSA was used as a model protein for growth factors and demonstrated how CPH systems could also be utilized as controlled GF delivery scaffolds. It was shown that the negatively charged BSA could be released faster and in greater quantities, relative to the passive release observed, when a constant potential of −0.6 V was applied or with the application of a biphasic pulse. It was also seen that when a constant oxidative potential was applied, release was slower compared to passive release, indicating the tendency of the oxidative state to withhold the charged BSA. This study demonstrated a significant push for CPHs as delivery systems by incorporating and releasing a large protein (66.4 kDa) rather than the typical small drugs (<1 kDa), which is challenging for conducting polymer systems. Li *et al.*, in an earlier study, demonstrated the electrically controlled release of heparin, an anticoagulating glycosaminoglycan, from PVA/PPy CPHs.^145^ These films were fabricated by first electropolymerizing pyrrole onto a conductive surface, then casting heparin-swollen PVA onto the PPy after aldehyde functionalization of the PPy surface. Although heparin was within the hydrogel and not the CP, electrical stimulation of the PPy with a constant 3.5 mA current yielded passive release of heparin from the hydrogel, two times higher than for CPH samples without stimulation. Another recent study utilized GelMA/PPy CPHs to release the neurotransmitter glutamate.^88^ Constant reduction of −0.6 V showed glutamate release from GelMA/PPy that was significantly greater than from PPy films alone (106.9 ± 7.5 *μ*g cm^−2^ vs 7.2 ± 1.59 *μ*g cm^−2^).

Ultimately, the use of CPHs in drug delivery is still a field in its infancy, where most studies still focus around proving and characterizing the release of pharmaceutical molecules or proteins and have not yet ventured into *in vitro* or *in vivo* applications. The primary purpose of the hydrogel phase is to provide a biocompatible physical environment that mimics that of the cell's natural environment. The benefits of this hydrogel phase for CPH DDS applications will, therefore, come to fruition when the field progresses toward *in vitro* and *in vivo* studies. The reason that the use of CPHs as DDSs is at an earlier stage of research compared to other DDSs is, indeed, it is a more novel system, and also because of added difficulties in achieving drug release from the CP with a hydrogel phase present. In simple terms, drug release from a CP-based system can be broken down into incorporation and release. The hydrogel phase can hinder incorporation of drugs into the CP by first acting as a barrier for the polymerization of the CP phase but also by acting as a barrier for the migration of the drug itself. Similarly, the hydrogel phase could act to prevent the release of drugs from the system. These factors add many variables to the release capabilities of the system, often yielding greatly inconsistent, reduced, or even totally suppressed release.

### C. Tissue engineering and biofabrication

The capability and adaptability of CPHs make them relevant to tissue engineering and biofabrication. It is particularly relevant to include CPs to electrically stimulate cells, or to release drugs from, while the hydrogel component ensures biocompatibility and overall mechanical properties. Combined, CPHs are paving the way for groundbreaking advancements in tissue engineering and biofabrication.^22,154,155^ This review further explores the use of CPH for electrically stimulating various types of mammalian cells.

#### 1. Cell responses to electrical stimuli

**FIG. 7.**
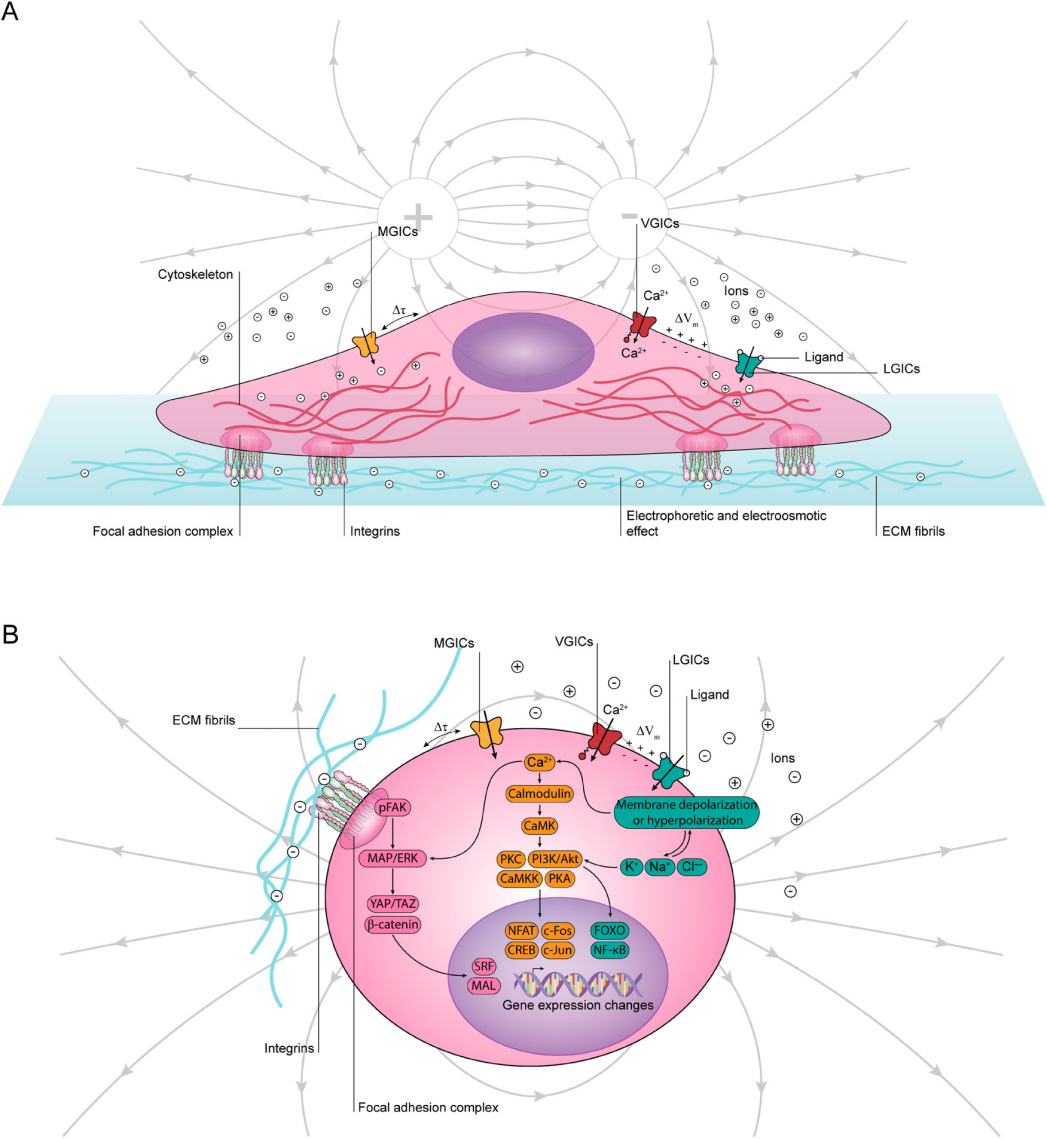
Cellular responses to electrical stimuli. (a) A schematic representation of a cell microenvironment and electric stimuli propagation via specific ion channels, identifying MGICs, VGICs, and LGICs as the major channels influenced by electrical stimuli. (b) Major intercellular signaling pathways involved in responses to electrical stimuli for MGICs, VGICs, LGICs, and integrin activation.

Cells perceive and respond to endogenous and exogenous electrical stimuli, a complex process involving various sensors and pathways leading to behavioral changes (Fig. 7). Endogenous direct currents, naturally occurring within the body, are integral to cellular functions. These currents arise from bioelectric phenomena, such as the differential distribution of ions across the cellular membrane.^156,157^ Exogenous electrical stimuli, either as direct or alternating currents, are externally applied and can mimic or modulate these natural electrical signals. These exogenous stimuli interact with the cell's natural bioelectrical environment, influencing cellular activities and functions.^156,157^

Figure 7 details the major pathways of electrical stimulus propagation in cultured cells. Based on the propagation mechanism, all cell types can be broadly categorized into three major groups: electrically excitable cells, electrically responsive (but non-excitable) cells, and electrically insensitive cells.^158^

Electrically excitable cells are characterized by their ability to rapidly respond to electrical stimuli trough specialized ion channels and generate an action potential transmitted through their membrane.^159^ These cells include neurons, muscle cells, such as cardiac and skeletal muscle, and certain endocrine cells, such as pancreatic α- and β-cells.^158,160^

Neurons are the most studied cells due to their capability of generation and propagation of an action potential, which is mainly associated with the activation of voltage-gated ion channels (VGICs), such as voltage-gated Na^+^ and K^+^ channels.^161^ The applied electric field initially interacts with different VGICs through the alteration of membrane potential (ΔV_m_).^162,163^ The change in ΔV_m_ prompts these channels to open, facilitating further ion exchange across the membrane [Fig. 7(a)]. The applied electric field can additionally induce changes in cell membrane tension (Δ*τ*), which results in the activation of various mechanically gated ion channels (MGICs).^164,165^ This leads to the influx of specific ions, such as Ca^2+^ or Na^+^, into the cell [Fig. 7(a)].

Muscle cells, especially cardiac cells, rely on electrical excitability for contraction and promoting heart rhythm. An action potential generation and propagation in these cells involve the activation of voltage-gated Na^+^ channels and L-type Ca^2+^ channels in cardiac muscle cells,^159^ ligand-gated ion channels (LGICs; e.g., nicotinic receptors), and voltage-gated Na^+^ channels in skeletal muscle cells,^166^ and voltage-gated or ligand-gated Ca^2+^ channels in smooth muscle cells.^167^ LGICs, typically activated by specific ligands or neurotransmitters, can be indirectly influenced by the electric field.^168,169^ Alterations in ΔV_m_ or local ion concentrations, which may occur due to electric field influence on other ion channels, can modulate the activity of LGICs, affecting their open or closed states [Fig. 7(a)].

In endocrine cells, voltage-gated Ca^2+^ channel activation results in ΔV_m_ propagation, leading to exocytosis.^160^

Electrically responsive cells cannot generate an action potential similar to electrically excitable cells and include different cell types, such as mesenchymal stem cells (MSCs) and osteoblasts.^158^ Electrical stimulation of these cells typically triggers intercellular Ca^2+^ signaling, which initiates the activation of specific transcription factors.^170^

Finally, electrically insensitive cells represent particular cell types, such as epithelial cells and adipocytes, lacking the specialized ion channels, which allow the rapid response of other electrically excitable or responsive cells to the applied electrical field. These cells are typically affected by the indirect impact of the applied electrical field or directly by electric fields of large magnitude.^171^ In such cells, integrin receptors, forming a crucial link with the ECM, can be involved in cellular responses to the applied electric field of higher magnitudes, which exerts electrostatic forces (F_e_) on negatively charged ECM proteins, influencing integrin receptors [Fig. 7(a)].^172^ While the direct effects of F_e_ on integrins are less well-established, combining these forces with changes in Δ*τ* and ECM configuration may alter the conformation and interaction of integrin receptors with ECM proteins. Moreover, changes in ΔV_m_ induced by the electric field could additionally impact integrin behavior.^34^

The signaling pathways, which propagate inwardly through the cell, are illustrated in Fig. 7(b). During activation of MGICs and VGICs, the movement of ions, particularly Ca^2+^, triggers a cascade of cellular signaling pathways. For instance, the influx of Ca^2+^ activates calmodulin, a Ca^2+^-binding messenger protein. This activation can stimulate various enzymes, including Ca^2+^/calmodulin-dependent protein kinase (CaMK). CaMK influences different downstream signaling molecules, which in turn affects enzymes like protein kinase A (PKA), protein kinase C (PKC), and Ca^2+^/calmodulin-dependent protein kinase kinase (CaMKK). As a result of Ca^2+^ signaling, transcription factors such as the nuclear factor of activated T-cells (NFAT) and cAMP response element-binding protein (CREB) are activated, leading to the regulation of gene expression.^173–175^ Transcription factors (c-Fos and c-Jun) that are generally associated with the outside-in mitogen-activated protein kinases/extracellular signal-regulated kinase (MAPK/ERK) signaling pathway, can be indirectly influenced by Ca^2+^ signaling due to the integration of multiple cellular pathways.^176^ VGICs, responding to ΔV_m_ changes, primarily affect cellular excitability and can indirectly influence the MAPK/ERK pathway.^177,178^ LGICs, modulated indirectly by the electric field, can impact signaling cascades such as the phosphoinositide 3-kinase/protein kinase B (PI3K/Akt) pathway.^179,180^ This pathway activates transcription factors like forkhead box O (FOXO) and nuclear factor kappa-light-chain-enhancer of activated B cells (NF-κB), leading to changes in gene expression.^181,182^ Additionally, mechanical stress caused by the electric field can alter integrin receptor configuration and interaction with ECM proteins. Integrin activation can lead to the autophosphorylation of focal adhesion kinase (pFAK), initiating mechanotransduction through the MAPK/ERK pathway and activating downstream molecules like Yes-associated protein/transcriptional coactivator with PDZ-binding motif (YAP/TAZ), β-catenin, serum response factor (SRF), and megakaryocytic acute leukemia (MAL), resulting in gene expression alterations.^183–186^ The overlap between signaling pathways activated via mechanical and electrical stimulation highlights potential synergies, that could be explored via conducting polymer hydrogel systems.

#### 2. Artificial muscles

**FIG. 8.**
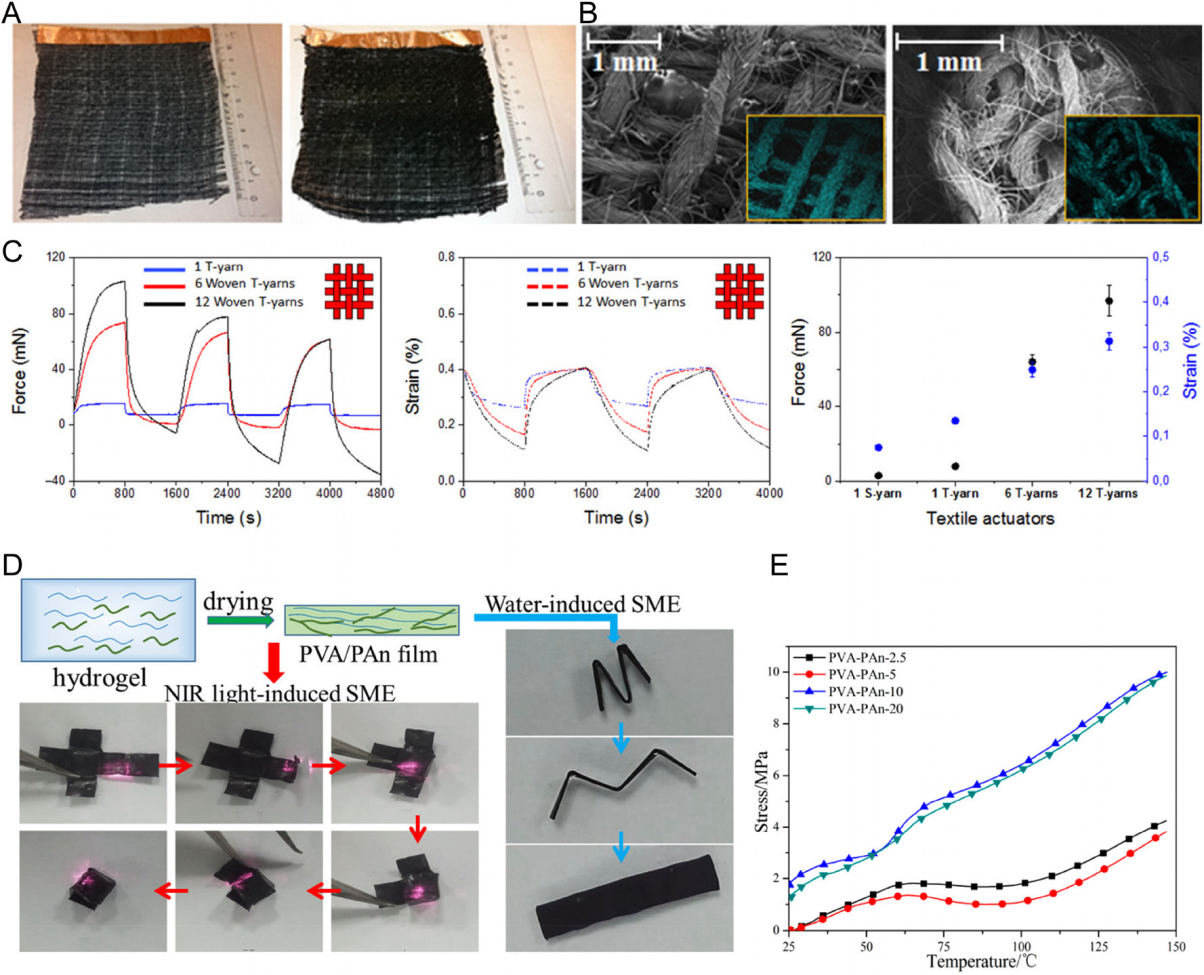
CP-based artificial muscles. (a) Photographs of PEDOT-coated (2.0 wt. %) and PEDOT-PPy-coated cellulose-based textuators. (b) Scanning electron micrographs of PEDOT-PPy-coated cellulose-based textuators. Energy-dispersive x-ray spectroscopy (EDX) sulfur maps are represented in the insets. (c) Electromechanical characterization of the woven textuators. Reproduced with permission from Maziz *et al.*, Sci. Adv. **3**(1), e1600327 (2017). Copyright 2017 Authors, licensed under a Creative Commons Attribution 4.0 License (http://creativecommons.org/licenses/by-nc/4.0/)/AAAS.^190^ (d) Representative photographs of a shape memory of PVA-PAn based composites. (e) Stress-temperature recovery behavior of PVA-Pan composites under 100% strain. Reproduced with permission from Bai *et al.*, ACS Appl. Mater. Interfaces **10**(16), 14017–14025 (2018). Copyright 2018 American Chemical Society.^191^

The advancement of skeletal muscle prosthetics has been remarkable, with continuous innovations aimed at replicating the unique characteristics of human muscle. A key focus in this field has been to identify materials that imitate muscle properties and ensure biocompatibility and functional efficacy. By combining the electrical conductivity inherent to CPs with the flexibility and biocompatibility of hydrogels, CPHs present a promising material for the development of next-generation muscle prosthetics. The role of CPHs in skeletal muscle prosthetics is particularly relevant when emulating the dynamic behavior of muscles. Muscles naturally expand and contract in response to electrical stimuli, a feature that is challenging to replicate in artificial systems.^187,188^ CPHs enable this crucial functionality with their inherent electrical conductivity and can be engineered to undergo controlled expansion and contraction, closely mimicking the natural movement of muscles (Fig. 8).^146,189^

Like many fields where CPHs have found use, research using CPs is a predecessor and has laid the foundations for more sophisticated, biocompatible CPH systems. For instance, Jager's group has been focusing on the development of artificial muscle “textuators.” These devices, created using a cellulose-based fabric coated with a PEDOT/PPy conductive layer, can exert an isometric force of 99 ± 8 mN and an isotonic strain of 0.3%. This is significant as it demonstrates the potential of CPs in replicating the mechanical aspects of skeletal muscles when electrically stimulated [Figs. 8(a)–8(c)].^190^ In another study, Da Silva and Oréfice designed poly(NIPAM-co-AAc)-based electrochemical actuators, incorporating chemically polymerized PANI. The resulting materials demonstrated thermo- and electrically responsive behavior, generating forces ranging from 0.4 to 3 mN.^192^ Pattavarakorn *et al.* developed polythiophene (PTh)-based CPHs by incorporating PTh into chitosan/carboxymethylchitosan (PTh/CS/CMCS) hydrogels to create electroactive artificial muscles. Their research demonstrated that the hydrogel could bend in response to an applied electric field, with the degree of bending depending on the field's strength. However, the incorporation of PTh led to reduced performance due to increased rigidity.^193^ Simaite *et al.* introduced polyvinylidene fluoride (PVDF)-graft-poly(ethylene glycol) monomethyl ether methacrylate (PEGMA) hydrogels incorporating PEDOT:PSS. The resulting PEDOT:PSS/PVDF-graft-PEGMA/PEDOT:PSS actuators, produced through robust solution casting, exhibited significant strains of 0.6% when subjected to an electric field of 1.5 V at 0.1 Hz. Notably, these CPH-based artificial muscles maintained their actuation capability even after 150 h of continuous actuation cycles.^194^ More recently, Bai *et al.* investigated PANI incorporating PVA-based (PVA-PAn) composites [Fig. 8(d)]. These materials demonstrated substantial tensile strength over 83 MPa, as well as recovery strength over 6 MPa [Fig. 8(e)].^191^

In summary, CPHs emerged as promising materials toward artificial muscle development, displaying pronounced mechanical properties and electroactive behavior.

#### 3. Skeletal muscle regeneration

**FIG. 9.**
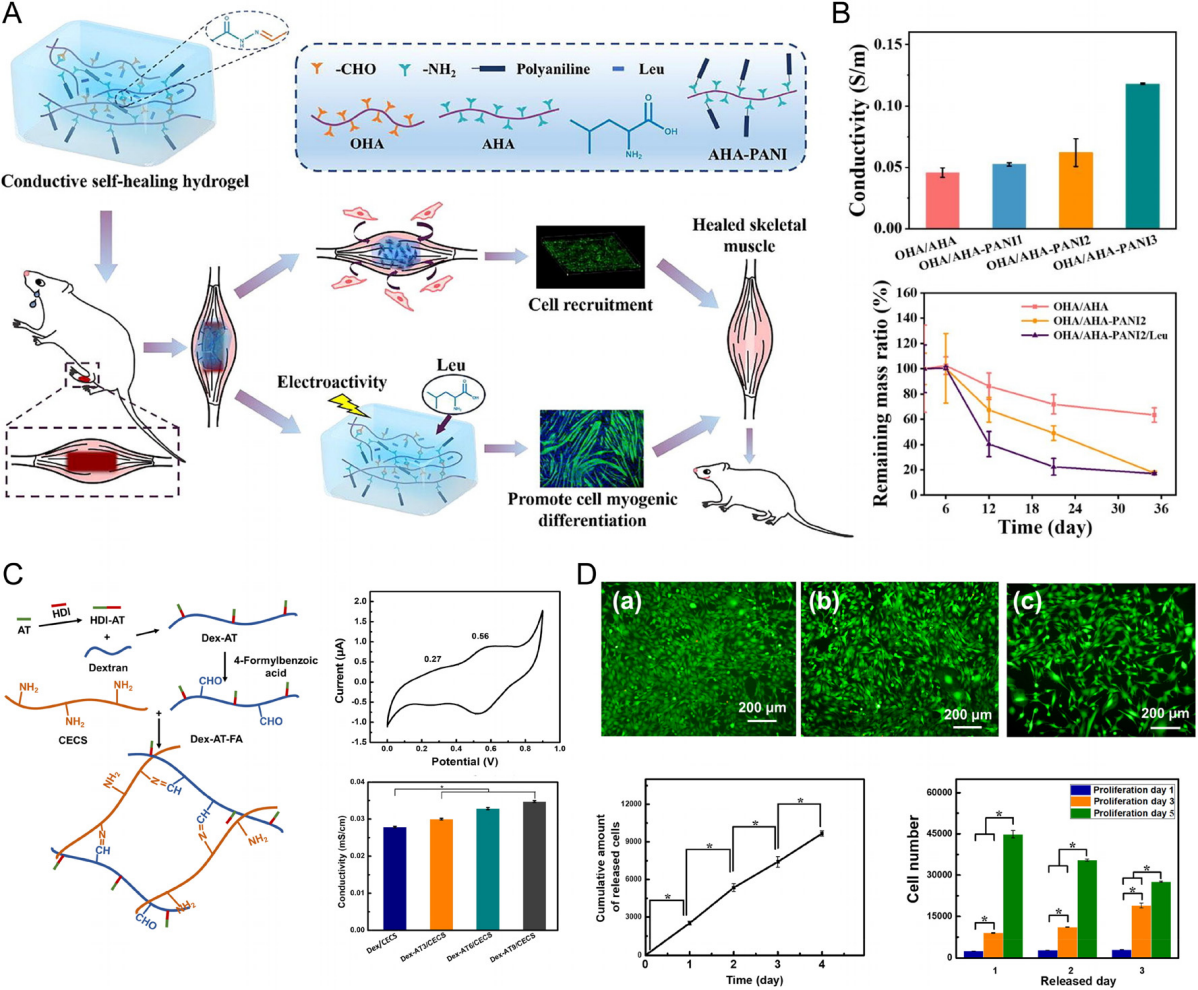
CP-based materials in skeletal muscle regeneration. (a) A schematic representation of hyaluronic acid-grafted-PANI (HA-PANI) hydrogel synthesis and implantation. (b) HA-PANI hydrogels demonstrated significant conductivity and promoted tibialis anterior muscle restoration *in vivo*, displaying significant biodegradability 4 weeks post-implantation. Reproduced with permission from Shi *et al.*, Chem. Eng. J. **457**, 141110 (2023). Copyright 2023 Elsevier.^195^ (c) A schematic representation of Dex-AT-FA/CECS hydrogel fabrication and its electrochemical properties. (d) C2C12 myoblast release from Dex-AT-FA/CECS hydrogels during 3 days of culturing. Reproduced with permission from Guo *et al.*, Acta Biomater. **84**, 180–193 (2019). Copyright 2019 Elsevier.^196^

Apart from artificial muscles, CPHs demonstrated pronounced performance in a skeletal muscle restoration, promoting differentiation of skeletal muscle precursor cells and improving muscle regeneration after injury (Fig. 9).^168,169^

Shi *et al.* designed a biodegradable, self-healing hyaluronic acid-grafted-PANI (HA-PANI) hydrogel with impressive electrical conductivity (6.21 × 10^−2^ S m^−1^). This conductivity is crucial for electrical stimulation in muscle tissues. The hydrogel not only improved the recruitment and differentiation of C2C12 myoblasts *in vitro* but also showed potential in promoting skeletal muscle regeneration *in vivo*, as seen in a rat tibialis anterior muscle defect model [Figs. 9(a) and 9(b)].^195^ Furthermore, Khalili *et al.* explored self-actuating multilayered scaffolds (SAMS) for muscle regeneration. These scaffolds, made from a flexible PEDOT:PSS with graphene oxide (PEDOT:PSS/GO) electrode and poly(ethylene glycol) diacrylate:acrylic acid (PEGDA:AA) electroactive layer, exhibited significant angular movement and conductivity (55 × 10^2^ S m^−1^), which are both vital for muscle contractility and cell survival. This study underlines the dual functionality of these materials in mechanical support and electrical stimulation for muscle tissue engineering.^197^ Guo *et al.* synthesized injectable electroactive dextran-graft-aniline tetramer-graft-4-formylbenzoic acid and N-carboxyethyl chitosan (Dex-AT-FA/CECS) hydrogels, which further emphasize the role of electrical properties in muscle tissue engineering [Fig. 9(c)]. These hydrogels were found to self-heal and exhibit some level of conductivity (2.7–3.4 × 10^−3^ S m^−1^), which is important for electrical stimulation. Additionally, these hydrogels were able to encapsulate and release cells, which highlights their potential to deliver cellular therapies alongside electrical stimulation for muscle repair and regeneration [Fig. 9(d)].^196^

In summary, these examples demonstrate the critical role of electrical stimulation in muscle tissue engineering whether through actuation, as seen in artificial muscle devices, or through direct electrical stimulation of cells, as observed in hydrogels and scaffolds.

#### 4. Cardiac regeneration

Electrical impulses govern the heart's ability to contract synchronously, and any disruption in this electrochemical environment can lead to severe cardiac dysfunction. The speed at which these electrical impulses, or conduction velocity, travel through the heart is crucial for maintaining this synchronous contraction. A proper conduction velocity ensures that all parts of the heart muscle contract in a coordinated manner, allowing the heart to pump blood efficiently. The average conduction velocity values range around 1.0–1.2 m s^−1^ for atrial myocardium, 0.02–0.05 m s^−1^ for atrioventricular node, and 1.2–4 m s^−1^ for bundle of His and Purkinje fibers.^198^ Any alteration in this velocity can lead to arrhythmias, where the heart's rhythm becomes irregular, compromising its function.

**FIG. 10.**
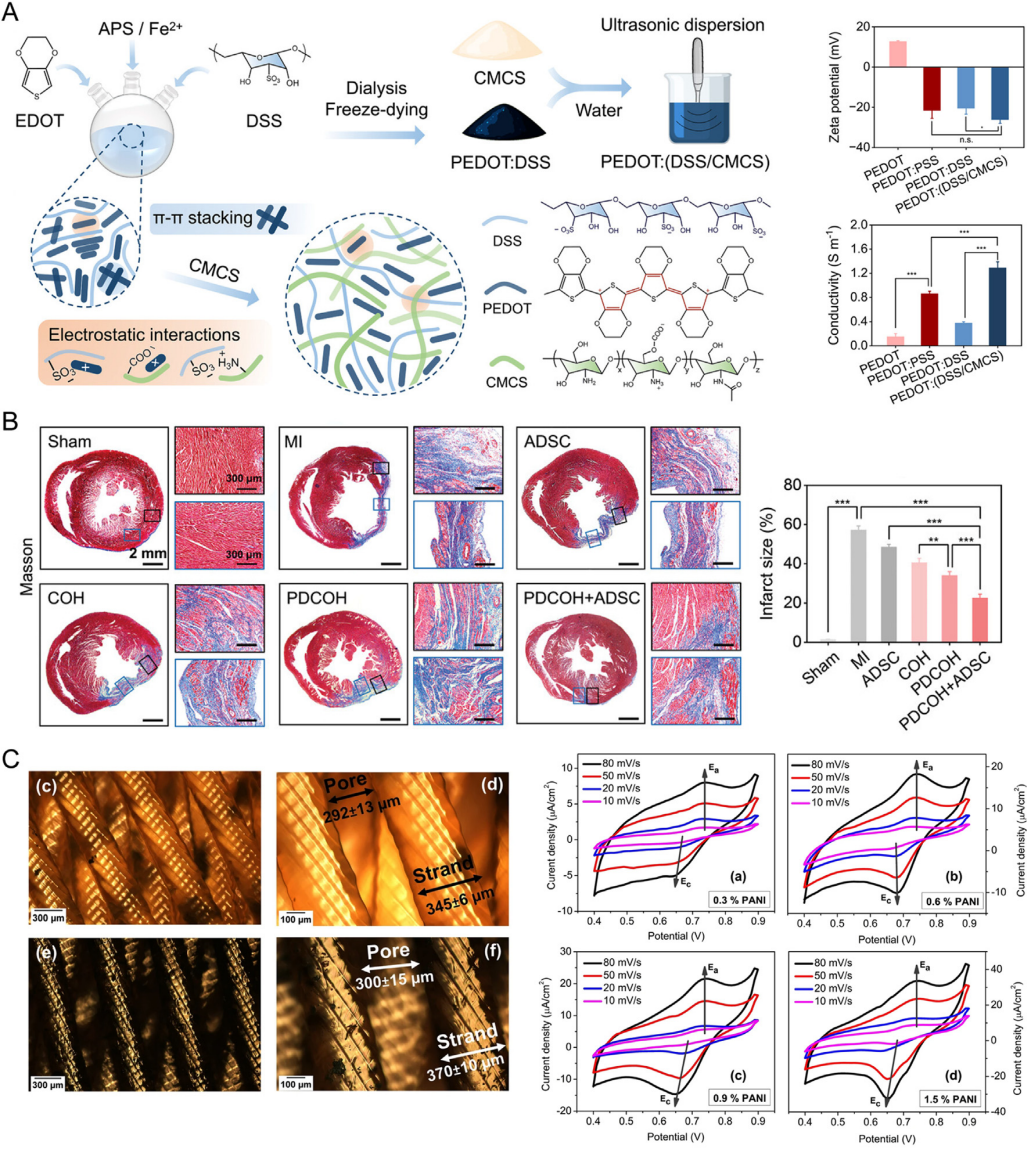
CP-based materials in cardiac regeneration. (a) A schematic representation of PEDOT:(DSS/CMCS) hydrogel fabrication and its conductive properties. (b) Differently modified PEDOT:(DSS/CMCS) promoted ventricular remodeling and collagen deposition, as well as resulted in the increase in thickness of the left ventricle and the decrease in the infarct size. Reproduced with permission from Yu *et al.*, Adv. Funct. Mater. **33**(15), 2211023 (2023). Copyright 2023 John Wiley and Sons.^208^ (c) Micrographs of 3D-printed electrically conductive scaffolds and CVs, representing their electrochemical activity depending on the PANI content. Reproduced with permission from Ul Haq *et al.*, Sci. Rep. **13**(1), 2863 (2023). Copyright 2023 Authors, licensed under a Creative Commons Attribution (CC BY) license.^209^

Early studies have previously shown that biomaterials, such as chitosan, alginate, collagen, and fibrin, could be used in cardiac repair;^199^ however, these scaffolds simply satisfy the mechanical and biocompatible criteria for such scaffolds and are non-conductive. The design of materials for cardiac repair necessitates a balance between both mechanical properties and electrical conductivity, with heart tissue also being mechanically dynamic and relying on efficient electrical signal propagation.^200^ When the scaffold does not act as an electrical bridge between the damaged site and the healthy cardiac tissue, arrhythmias can commonly arise.^201,202^ CPHs, with their inherent electrical conductivity, offer the ability to maintain and regulate this electrical environment, thus ensuring proper heart functioning. CPHs, which combine the advantageous properties of CPs with the highly hydrated network structure of hydrogels, offer a solution that meets both the mechanical and electrical criteria. Their electrical conductivity is vital for replicating the natural electrochemical environment of the heart and is essential for the synchronized contraction of cardiomyocytes. Simultaneously, the hydrogel component provides a biocompatible scaffold that supports cell adhesion, proliferation, and differentiation while also being capable of delivering bioactive molecules for tissue regeneration (Fig. 10).^202–205^

A series of studies illustrate the diverse and impactful applications of CPHs in cardiac repair. For instance, Mihic *et al.* developed CPHs composed of PPy grafted onto chitosan (PPy-chitosan), which enhanced calcium signaling and conduction velocity in the scarred region to the healthy tissue values, which led to improved outcomes post-myocardial infarction. This demonstrates the potential of CPHs in enhancing heart function through improved electrical signaling.^206^ Further emphasizing the versatility of CPHs, Wei *et al.* introduced HIF-1α releasing tetraaniline and partially oxidized alginate (ALG-CHO-TA) based hydrogels that facilitate targeted therapeutic delivery to promote vascularization in tandem with electrical signaling possible from the ALG-CHO conductive composite phase.^207^ More recently, Yu *et al.* developed bioinspired CPHs based on PEDOT:(dextran sulfate/carboxymethyl chitosan) [PEDOT:(DSS/CMCS)], which showed significant conductivity reaching up to 1.3 S m^−1^ [Fig. 10(a)]. Their materials demonstrated an ability to adapt to changes in cardiac tissue, effectively mimicking the networks of Purkinje fibers and significantly reducing the QRS interval duration from approximately 70 ms in myocardial infarction animals to 20.3 ± 1.6 ms in animals treated with PEDOT-based hydrogel, marking a crucial advancement in cardiac repair materials [Fig. 10(b)].^208^

Studies have also utilized 3D printing to mimic cardiac fiber alignment better and produce more realistic cardiac tissue models. Haq *et al.* utilized 3D printing micro-stereolithography to create electrically conductive scaffolds mimicking myocardial fiber alignment, illustrating the innovative use of technology in developing CPHs [Fig. 10(c)]. These scaffolds supported cardiac progenitor cell survival, indicating their potential in cardiac tissue engineering.^209^ Another work related to the use of 3D printing conducted by Zhou *et al.* investigated bi-continuous conducting polymer hydrogel (BC-CPH) based on PEDOT:PSS and hydrophilic polyurethane for bioelectronic interfaces, achieving high conductivity (over 11 S m^−1^) and stretchability. 3D printing allowed the rapid fabrication of tissue-like interfaces, demonstrating long-term electrophysiological recording capabilities when implanted in rat hearts. This work exemplifies the integration of CPHs with electronic interfaces, expanding their potential use in cardiac repair.^210^

In summary, these studies collectively highlight the significant role of CPHs in cardiac tissue regeneration. By combining structural support, electrical interaction, and the capability for therapeutic delivery, CPHs stand out as a comprehensive solution for heart repair, effectively bridging the gap between artificial materials and the dynamic requirements of cardiac tissue.

#### 5. Epithelial regeneration and wound healing

**FIG. 11.**
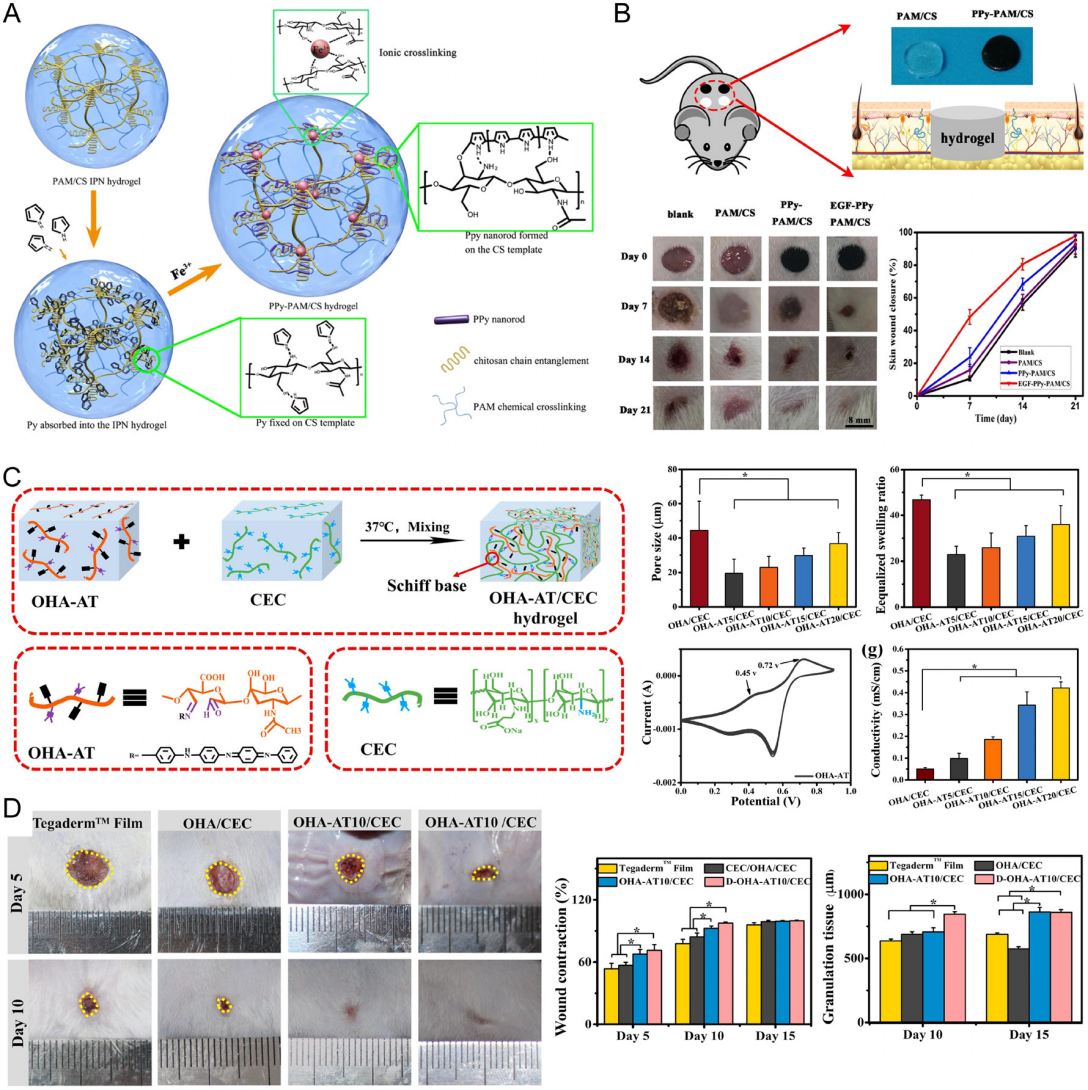
CP-based materials in epithelial regeneration. (a) A schematic representation of PPy–PAM/CS hydrogel fabrication. (b) PPy–PAM/CS hydrogels enhanced the wound healing rates for repairing full-thickness defects in rats. Reprinted/adapted with permission from Gan *et al.*, ACS Appl. Mater. Interfaces **10**(42), 36218–36228 (2018). Copyright 2018 American Chemical Society.^152^ (c) A schematic representation of OHA-AT/CEC hydrogel preparation, as well as its mechanical properties and conductivity. (d) Photographs representing wound healing dynamics in rats 10 days post-implantation. OHA-AT/CEC hydrogel transplantation promoted faster wound contraction rates and granulation tissue formation in rats *in vivo.* Reproduced with permission from Qu *et al.*, Chem. Eng. J. **362**, 548–560 (2019). Copyright 2019 Elsevier.^216^

Epithelial cells are among the most abundant of cells within the skin, blood vessels, and organ linings. Epithelial tissues play a significant role in our bodily functions, including protection from the environment, secretion, absorption, excretion, and more. Their direct exposure to the environment does make epithelial tissues highly prone to wound formation, and the design of scaffolds to aid in wound healing is currently a paramount area within tissue engineering. Epithelial tissue transports ions on the epidermis, forming an “epidermal battery,” and when the epithelial tissue is broken, this transmembrane potential is disrupted.^211^ CPHs are particularly effective in wound healing because they can provide conductive pathways that mimic that of uninjured epithelial tissues.^212^ These electrical signals play a pivotal role in wound healing by also enhancing cellular behaviors, such as cell migration, a vital step where cells move toward the wound site. Furthermore, these signals stimulate cell proliferation, where increasing cell numbers helps to restore lost or damaged cells at the wound site. Additionally, electrical signals influence cell differentiation, guiding cells to mature into specific types needed for effective wound repair, such as keratinocytes for skin regeneration.^213^ Beyond their conductive capabilities, CPHs offer a three-dimensional porous structure, which is beneficial for several critical aspects of wound healing, including efficient gas exchange, nutrient diffusion, and waste removal. By integrating these properties, CPHs act as a physical scaffold for new tissue growth and actively participate in the healing process, potentially reducing healing time and improving the quality of the newly formed epithelia (Fig. 11).^214,215^

While CPHs are less commonly used in epithelial repair compared to cardiac regeneration, they offer significant benefits in this area, particularly due to their ability to facilitate electrical signal transfer, which aids in epithelial tissue regeneration.^172,217^ Gan *et al.* developed a novel hydrogel using a PAAm/chitosan interpenetrating network with PPy nanorods (PPy–PAM/CS) created through FeCl_3_ *in situ* polymerization [Fig. 11(a)]. This hydrogel demonstrated substantial mechanical strength and conductivity of 0.3 S m^−1^. Notably, it showed a controlled release of dexamethasone and enhanced skin wound healing in rats, including blood vessel and hair follicle formation, 21 days post-implantation [Fig. 11(b)].^152^ Qu *et al.* created CPHs consisting of N-carboxyethyl chitosan and oxidized hyaluronic acid-graft-aniline tetramer (OHA-AT/CEC). These hydrogels displayed stable mechanical properties, high swelling ratio, appropriate gelation, biodegradation time, and free radical adsorption [Fig. 11(c)]. With a conductivity of 0.042 S m^−1^, they significantly promoted wound healing, tissue remodeling, and angiogenesis [Fig. 11(d)].^215^ Bhattacharjee and Ahearne fabricated transparent electroconductive silk fibroin/PEDOT:PSS scaffolds for corneal epithelial reconstruction. These materials showed reduced impedance with increased PEDOT:PSS concentration, supporting human corneal epithelial cell survival and functioning.^218^

In diabetic foot ulcer management, Liu *et al.* introduced CPHs comprising poly(tannic acid)-doped PPy nanofibrils in a PAAm-acrylated adenine network [P(Py-TA)/CHA]. These hydrogels, with a conductivity of 0.18 S m^−1^, displayed transparency, adhesiveness, and hemostatic properties. They enhanced hemostasis, tissue remodeling, angiogenesis, and reduced inflammation in rat models.^219^ Zhao *et al.*^220^ fabricated a supramolecular assembly of polydopamine-decorated silver nanoparticles, polyaniline, and polyvinyl alcohol (PDA@AG NPs/CHP) as epidermal sensors and diabetic foot wound dressings. The resulting hydrogel possessed tunable mechanical and electrochemical properties and demonstrated full wound closure on rat feet over 20 days, during which time control wounds had insignificantly healed compared to a control group. They also show the antibacterial properties of the system, which encapsulates the modern, multifaceted approach to wound healing dressings, wherein single treatment methods are often suboptimal, and efforts should be made toward preventing inflammation. More recently, Wang *et al.* developed an all-in-one self-powered CPH using a sodium hyaluronate-based hydrogel and PEDOT:PSS, freeze–thawed to create a supercapacitor. The CPHs exhibited an ionic conductivity of 1.27 S m^−1^, suitable mechanical properties, high water absorption capacity, and biocompatibility. Notably, these wound dressings enhanced skin wound healing in rats, including epithelial structure restoration and blood vessel formation. The integrated supercapacitor allowed for self-powered electrical stimulation, eliminating the need for an external power source.^221^

These examples illustrate the diverse applications and effectiveness of CPHs in epithelial repair, demonstrating their potential as innovative materials in wound healing and tissue regeneration.

#### 6. Bone and cartilage regeneration

**FIG. 12.**
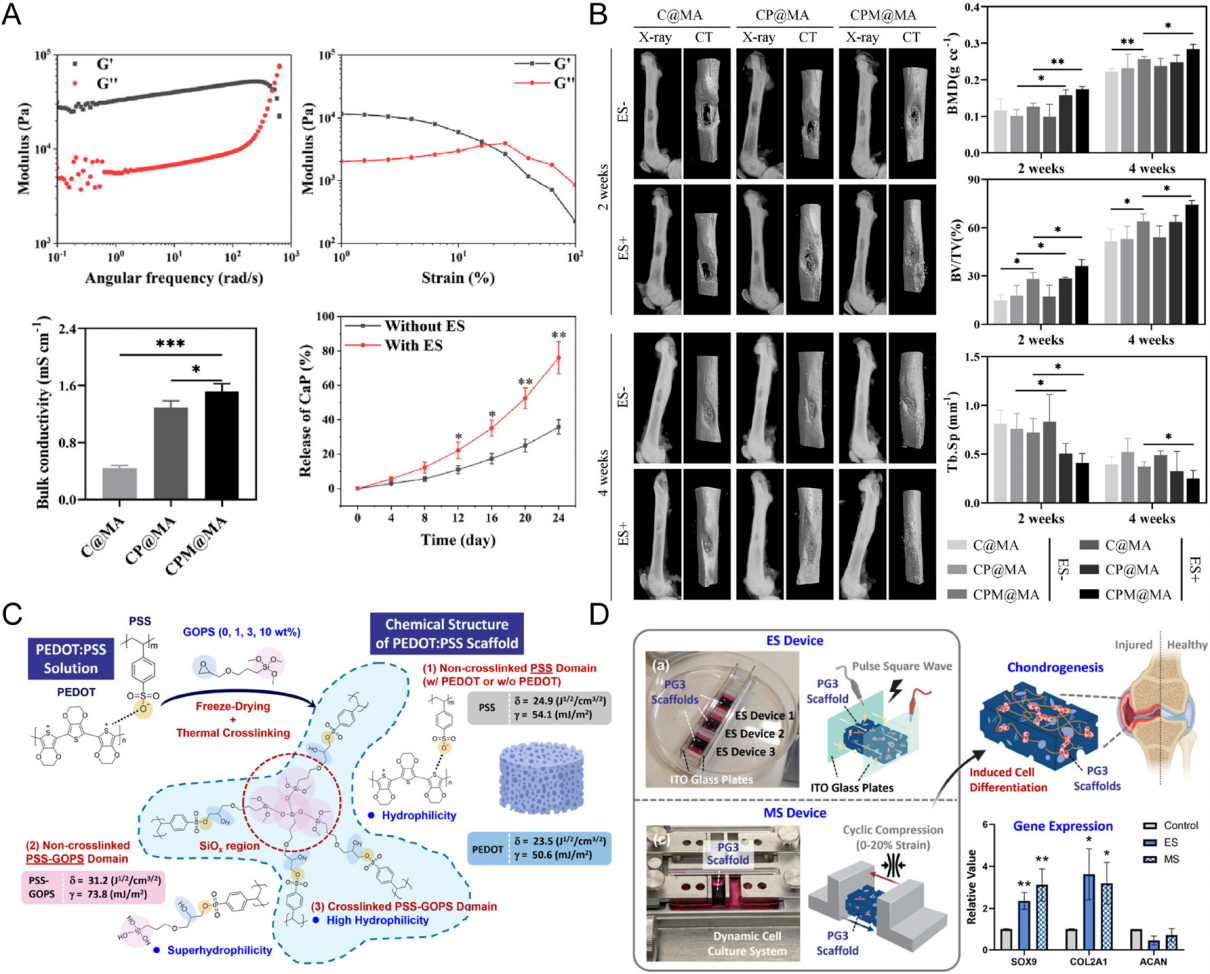
CP-based materials in bone and cartilage regeneration. (a) Mechanical properties of CPM@MA CPHs. (b) CPM@MA hydrogels restore critical femoral defect, improving bone mineral density (BMD), bone volume/tissue volume ratio (BV/TV), and trabecular spacing (Tb.Sp) 4 weeks post-implantation in rats. Reproduced with permission from Yu *et al.*, Biomaterials **301**, 122266 (2023). Copyright 2023 Elsevier.^224^ (c) A schematic representation of PEDOT:PSS-based CPH fabrication. (d) PEDOT:PSS-based CPH platform for studying the simultaneous impact of electrical and mechanical stimuli on encapsulated cells. SOX9 and COL2A1 gene expression is up-regulated in human adipose stem cells cultured inside the designed CHPs. Reprinted/adapted with permission from Liu *et al.*, Biomacromolecules **24**(8), 3858–3871 (2023). Copyright 2023 American Chemical Society.^225^

The electrical properties of CPHs are particularly advantageous for bone and cartilage regeneration, as they facilitate specific types of cellular stimulation essential for tissue growth and repair. This includes the promotion of osteoblast proliferation, essential for bone formation; chondrocyte maturation, crucial in cartilage development; and the synthesis of specific ECM components that are key to the structural integrity of these tissues. The stimulation provided by CPHs often involves electric fields or currents that can influence cell behavior and tissue formation. Additionally, the physicochemical versatility of CPHs enables the encapsulation and precise release of bioactive molecules and growth factors. This targeted and temporally controllable delivery is critical for supporting tissue repair and integration, ensuring these therapeutic agents are available at the right time and place in the healing process. The gel properties desired in CPHs include biocompatibility, appropriate mechanical strength to support the skeletal system, and a porous structure for efficient nutrient and molecule transport. CPHs are preferred over simple CPs due to their hydrogel component, which adds crucial characteristics like high water content, resembling the natural environment of tissues. This makes them more biocompatible and less likely to cause adverse reactions in the body, and this is why more recent studies have emphasized the exploration of CPHs. The hydrogel tissue interface also allows for better integration with the surrounding tissue, providing a more supportive and natural scaffold for cell growth and tissue development (Fig. 12).^222,223^

In tissue engineering and biofabrication, particularly for bone and cartilage regeneration, the ideal material should possess specific properties to mimic and support the native tissues. For bone tissue, materials should offer mechanical strength, osteoconductivity to support cell attachment and bone formation, and a porous structure for nutrient flow and cell migration. For cartilage, elasticity, resistance to compressive forces, and a smooth surface to facilitate low-friction movement are essential.^157^

CPHs are being explored in this context, as their electrical properties can stimulate cellular activities crucial for tissue growth and repair. For instance, in bone regeneration, materials like the PANI nanoparticles and polylactide (PANI/PLA) composites developed by Chen *et al.* demonstrate essential features like porosity and conductivity (3.2 S m^−1^), promoting osteogenic gene expression and facilitating calcium deposition. These characteristics are vital for supporting the metabolic activity of mesenchymal stem cells and enhancing bone formation.^226^ Similarly, Yu *et al.* introduced a nano-conductive osteogenic hydrogel composed of calcium phosphate-PEDOT:PSS-magnesium titanate-methacrylated alginate (CPM@MA) hydrogel for bone regeneration, which underlines the significance of conductivity (up to 15.2 ± 0.9 × 10^−2^ S m^−1^), biocompatibility, and osteoinductivity in materials designed for bone healing [Figs. 12(a) and 12(b)].^224^

In cartilage regeneration, CPH properties can be leveraged to support chondrogenic differentiation. For example, Prasopthum *et al.* found that 3D-printed CPHs based on tetraaniline-b-polycaprolactone-b-tetraaniline (TPT) facilitate chondrogenic differentiation of chondroprogenitor cells, highlighting the potential of CPHs in promoting cartilage repair.^227^ Keate *et al.* introduced collagen sponges with PEDOT-sulfonic acid sodium salt (S) to create a PEDOT-S-collagen (PEDOT-ACS) composite, which also demonstrates this potential by maintaining cell proliferation and enhancing glycosaminoglycan production, crucial for cartilage matrix formation.^228^ Furthermore, Liu *et al.* developed a PEDOT:PSS-based CPHs for articular cartilage restoration, capable of applying cyclic compressive forces and electrical stimulation, representing an advanced approach to simulating the natural cartilage environment, promoting chondrogenic differentiation effectively [Fig. 12(c) and 12(d)].^225^

These examples illustrate how the unique properties of CPHs, such as conductivity, biocompatibility, and mechanical strength, can be tailored to meet the specific requirements of bone and cartilage regeneration, offering promising strategies for the treatment of skeletal system injuries and degenerative diseases.

#### 7. Neural regeneration

**FIG. 13.**
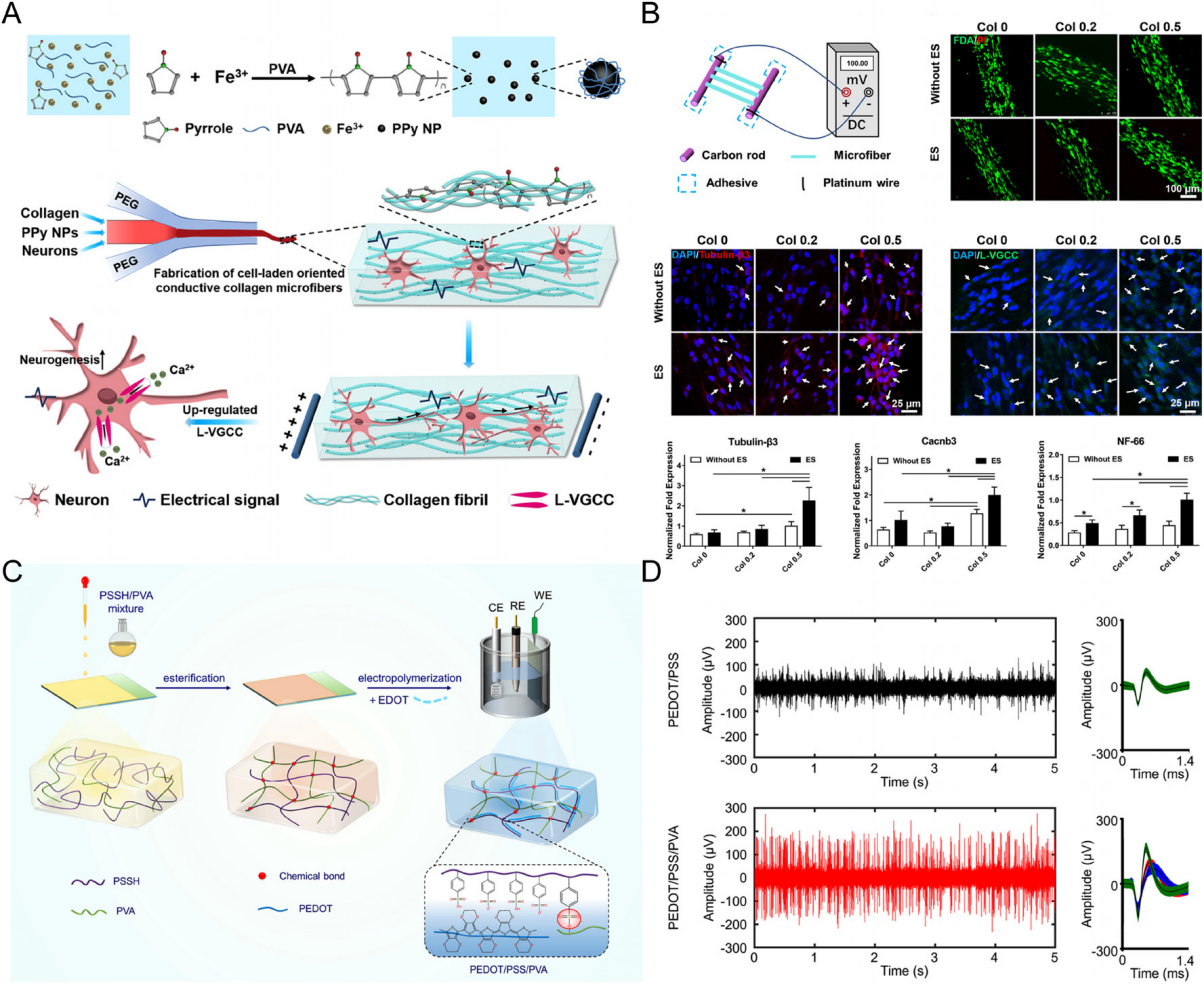
CP-based materials in neural regeneration. (a) A schematic representation of Collagen/PPy CPH fabrication and the development of cell-laden PPy-incorporated collagen hydrogel microfibers. (b) A schematic representing externally stimulated Collagen/PPy CPHs. The incorporation of PPy into the microfibers promoted neurogenesis in PC12 cells, while electric stimulation further enhanced this effect, particularly in samples with a higher concentration of PPy. Reprinted/adapted with permission from Wu *et al.*, ACS Appl. Mater. Interfaces **11**(25), 22152–22163 (2019). Copyright 2019 American Chemical Society.^235^ (c) PEDOT:PSS/PVA hydrogel preparation schematic representation. (d) Illustrative instances of unprocessed spike traces and the waveforms of neuronal units captured from electrodes treated with PEDOT:PSS and PEDOT:PSS/PVA 12 weeks post-transplantation in mice. Reprinted/adapted with permission from Yan *et al.*, ACS Appl. Mater. Interfaces **15**(35), 41310–41323 (2023). Copyright 2023 American Chemical Society.^235^

Materials and solutions that can enhance nerve regeneration are particularly important due to the relatively poor self-regenerative capacity of the central nervous system.^229^ Development of these systems comes with unique challenges, though, as limited blood circulation means that direct intrathecal implantation around the spinal cord is typically required.^230^ Hydrogel-based systems have become popular for this as their tailorable stiffness to that of neural tissue (<1 kPa) and ability for electrical signal transmission have been shown to improve the survival, differentiation, and expression of neural progenitor cells (NPCs). The matching of both conductivity and mechanical properties to that of the natural nervous tissues are critical for inducing repair, and CPHs can satisfy both. For conductivity, nerve tissue ranges between 0.08 and 1.3 S m^−1^,^231^ which is large compared to other tissues such as cardiac (0.2–0.5 S m^−1^)^232^ and skeletal muscle (0.8–4.5 × 10^−3^ S m^−1^).^196^ The Young's modulus of tissues in the central nervous system, and hence, the desired stiffness of mimetic therapeutic scaffolds is very low for both brain tissue (∼1 kPa) and spinal cord tissue (1.23 kPa).^233^ Naturally, CPH usage in nerve cell treatments has gained traction as they can preserve the soft hydrogel properties while expressing enhanced electrical capabilities (Fig. 13).^234^

Recent research has demonstrated how CPHs can be utilized to treat neurological degeneration or trauma. For instance, Xu *et al.* utilized CMCS as a biodegradable hydrogel network with chemically polymerized PEDOT within.^237^ *In vitro* cell studies were conducted on neuron-like rat phaeochromocytoma (PC12) cells, where the enhanced mechanical strength and conductivity provided by the PEDOT enhanced cell viability and proliferation. Collagen/PPy CPHs explored by Wu *et al.* promoted neurogenesis of PC12 cells with external electrical stimulation of 100 mV/cm for 1 h/day. Neurogenesis was found to be due to upregulated expression of an L-type voltage-gated Ca^2+^ channels in response to the electrical stimulation [Figs. 13(a) and 13(b)].^235^ Yan *et al.* constructed a PEDOT:PSS/PVA CPH [Fig. 13(c)] and cultured hippocampal neurons on that scaffold.^236^ Cultures of hippocampal neurons and PC12 cells on the scaffold revealed enhanced neurite outgrowth, indicating improved biocompatibility, in comparison with PEDOT:PSS films. Electrophysiological and histological examinations further verified the enhanced interface between electrode and neural tissue, leading to superior quality of recording signals [Fig. 13(d)]. Abidian *et al.* developed mechanically reinforced agarose nerve conduits that were made conductive with a layer of PEDOT to be used for axonal regeneration. These conduits were implanted in peroneal nerve gaps in rats, and axonal growth was encouraged by these conduits.^238^

#### 8. Neural interfaces and prosthetics

Neural interfaces act as a platform to connect neural tissues and electronic devices with the purpose of, for instance, tracking bioelectric signals, or providing electrical charges to the neural tissue,^239,240^ as well as promoting targeted cell attachment and ingrowth of neural tissue to the interface.^66^ CPHs are an emerging material choice for neural interfaces due to their ability to satisfy the required parameters for neural interface systems, such as good impedance, cathodic charge storage, charge injection capacity, and biocompatibility.^239^ Abidian *et al.* demonstrated the usage of CPHs as neural interfaces by developing a alginate/PEDOT CPH system with dexamethasone incorporated biodegradable nanofibers. The result was a low impedance, high charge density, and controlled releasing CPH system capable of acting as a compatible bridge between neural tissue and neural microelectrodes by stabilizing this electrode/tissue interface.^91^ More recently, Strakosas *et al.* developed a new method for utilizing flexible conducting materials and neural interfaces without a substrate. CPH precursors were injected into zebrafish brain, where enzymatic polymerization occurred to form the CPH with high conductivity, resulting in an extremely natural and seamless interface with the neural tissues with minimal damage.^241^

Spinal cord injuries (SCI) in a particularly relevant trauma to apply CPHs to. Current SCI treatment focuses on filling the injured cavity to promote the reconstruction of the microenvironment via neuronal differentiation.^22^ These therapies are only marginally effective, though, as mechanical and electrical mismatches between implanted material and the natural microenvironment are difficult to mimic.^22,242^ Implantable CPHs have been explored as promising materials to address these challenges for SCI therapy. Yang *et al.* tuned agarose/gelatin/polypyrrole (Aga/Gel/PPy, AGP3) CPHs to be similar in conductivity and modulus to the spinal cord by altering the concentrations of agarose and PPy.^243^ *In vitro* cultures showed excellent biocompatibility and differentiation of neural stem cells (NSCs) toward neurons. *In vivo* studies showed that the AGP3 scaffold promoted endogenous neurogenesis rather than glial fibrosis formation. Zhou *et al.* developed a tannic acid/PPy conductive hydrogel, which exhibited excellent electrical conductivity (5–18 S m^−1^) and mechanical properties (0.3–2.2 kPa, varied by altering TA concentration) to mimic spinal cord tissue.^244^ *In vitro* studies showed that the CPHs with higher conductivity accelerated the differentiation of NSCs into neurons while suppressing the development of astrocytes. *In vivo*, endogenous NSC neurogenesis was activated within the lesion area, resulting in the recovery of locomotive function.

#### 9. Ongoing challenges with CPH tissue engineering scaffolds

While CPHs show promise as regenerative scaffolds within tissue engineering due to their attractive properties of both ideal and tunable mechanical properties and electrical signal transmitting capabilities, some challenges remain and should be a focus of future research. For instance, there is a current lack of clinical and *in vivo* data to properly identify the ideal electrical parameters for different applications.^245^ Furthermore, for clinical applications, injectable CPHs are the most practical and common to administer; however, this method often makes it more challenging to reach the required mechanical strength and control the conductivity adequately.^22^ *In vivo* degradation of the hydrogel is also a challenge for many systems, which is essential because maintaining the mechanical stability of the CPH throughout the therapy is paramount.^22^ The CP component generally does not biodegrade, and the mismatch in degradation between the hydrogel and the CP is an additional challenge. Consideration needs to be given toward the cytotoxic implications of having free CP in the instance of hydrogel degradation, as although most CPs have been shown to be non-cytotoxic,^246–249^ the by-products of synthesis, such as the various oxidants and dopants used, could be.^250,251^ Ideally, the two polymer systems should be fully interpenetrated to harness the full benefits of combining CPs with hydrogels.^67^ Achieving interpenetration is a difficult task and depends greatly on the properties of the mixed polymer systems and the CP polymerization method used. While it is easier to accomplish through chemical polymerization since polymerization occurs with already dispersed monomers, chemically polymerized CPs are known to have diminished conductivity. This could be a particular issue when polymerizing the conducting polymer within an already formed gel, which may hinder the growth of long polymer, and connection between polymers. Composites that include other conductive elements (carbon nanotubes or metal particles/rods) as well as chemically polymerized CPs may offer additional benefits in terms of conductivity and interpenetration. For electrochemically polymerized CPs, interpenetration is rare within literature, although recent efforts have demonstrated promising partial penetration.^88,95,236^ A deeper understanding of how to achieve interpenetrating networks is required for CPHs to realize their proper potential within the field of tissue engineering, as this will aid in better optimizing the mechanical and electrical properties of designed systems, enhancing therapeutic function and efficacy.

Finally, CPH technology is still in a relatively infant stage, focusing mainly on research rather than production and application. Therefore, it currently lacks low-cost, high-scale, and controllable production means, which are essential for moving any engineered system toward real-world applications.^16^

## V. CONCLUSION

Conducting polymer hydrogels represent a unique class of materials that combines the remarkable properties of both its constituents. Within the field of biomedicine, as highlighted during this review, the properties of CPHs are uniquely suited for applications within biosensing, drug delivery, and tissue engineering. As we navigate the future of biomedicine, conducting polymer hydrogels hold immense promise as dynamic and multifunctional materials, poised to revolutionize therapeutic approaches and diagnostics. By fostering continued research, conducting polymer hydrogels will play an increasingly integral role in shaping the next generation of biomedical technologies, ultimately improving patient outcomes and advancing the frontiers of biomedicine.

## Data Availability

Data sharing is not applicable to this article as no new data were created or analyzed in this study.
